# Lipids and membrane-associated proteins in autophagy

**DOI:** 10.1007/s13238-020-00793-9

**Published:** 2020-11-05

**Authors:** Linsen Li, Mindan Tong, Yuhui Fu, Fang Chen, Shen Zhang, Hanmo Chen, Xi Ma, Defa Li, Xiaoxia Liu, Qing Zhong

**Affiliations:** 1 State Key Laboratory of Animal Nutrition, Ministry of Agriculture Feed Industry Centre, College of Animal Science and Technology, China Agricultural University, Beijing, 100193 China; 2 Key Laboratory of Cell Differentiation and Apoptosis of Chinese Ministry of Education, Department of Pathophysiology, Shanghai Jiao Tong University School of Medicine, Shanghai, 200025 China

**Keywords:** autophagy, membrane-associated proteins, membrane-associated biochemistry assays, ATG2, ESCRT, lipid transfer, elongation, scission, fusion

## Abstract

Autophagy is essential for the maintenance of cellular homeostasis and its dysfunction has been linked to various diseases. Autophagy is a membrane driven process and tightly regulated by membrane-associated proteins. Here, we summarized membrane lipid composition, and membrane-associated proteins relevant to autophagy from a spatiotemporal perspective. In particular, we focused on three important membrane remodeling processes in autophagy, lipid transfer for phagophore elongation, membrane scission for phagophore closure, and autophagosome-lysosome membrane fusion. We discussed the significance of the discoveries in this field and possible avenues to follow for future studies. Finally, we summarized the membrane-associated biochemical techniques and assays used to study membrane properties, with a discussion of their applications in autophagy.

## INTRODUCTION

**Figure 1:**
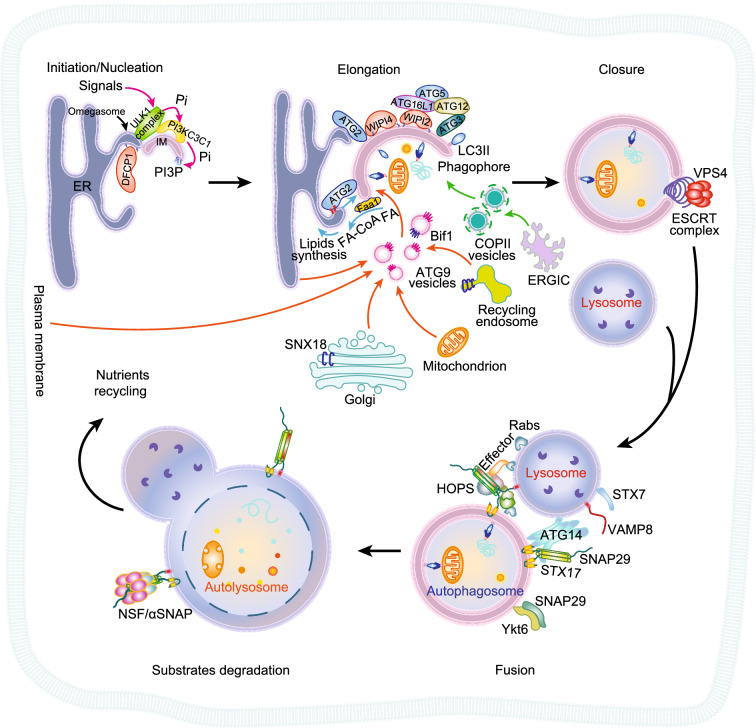
**Overview of autophagy with membrane-associated proteins highlighted**. Cells go through the following steps to complete a cycle of autophagy: 1) autophagy initiation (signals activate autophagy) and nucleation of the phagophore/isolation membrane (IM, another name of the phagophore); 2) phagophore elongation; 3) closure to form the autophagosome; 4) fusion between the autophagosome and lysosome; 5) degradation of substrates in autolysosomes. Autophagy begins when cells sense the stimulation signals. The omegasome (a PI3P-enriched subdomain of ER where DFCP1 localizes through binding to PI3P) is the platform for the nucleation of the phagophore. This step involves two important complexes, the ULK1 complex and the PI3KC3C1 complex. The ULK1 complex phosphorylates and activates the PI3KC3C1 complex. The activated PI3KC3C1 complex generates PI3P from PI. Then, PI3P recruits WIPIs, which in turn recruit more autophagy machinery proteins. ATG12~ATG5-ATG16L1 recruited by WIPI2 catalyze ATG3-mediated conjugation of ATG8 family proteins with membrane resident PE, generating products like LC3II, which is the characteristic signature of autophagic membranes and is involved in ATG9 vesicle sequestration of cargo. There are multiple membrane sources of the autophagosome, including ER, Golgi, mitochondria, endosome, ERGIC, and plasma membrane. There are few possible ways for lipid transport, including ATG9 vesicle-mediated transport, COPII vesicle-mediated transport, ATG2-mediated lipid transport, etc. The cargos are sequestered while the autophagosomal membrane expands. Then, the sealing of this membrane structure by scission proteins such as ESCRT and other regulators gives rise to a double-membrane structure called the autophagosome. After becoming fully sealed, the autophagosome will recruit tethering proteins and SNARE proteins for fusion. Once fused, the acidic hydrolases in the lysosome degrade the autophagic cargos, salvaged nutrients are released back to the cytoplasm to be used by the cells, and the cis-SNARE complex is disassembled and recycled by NSF/αSNAP complex

Autophagy is a process by which cells break down and recycle their components. Autophagy occurs at a basal level in all cells, but it can be upregulated during stress, starvation, or infection. Autophagy is essential for the maintenance of cellular homeostasis and its dysfunction has been linked to various diseases, including cancer, neurodegeneration, and immune diseases (Levine and Kroemer, 2019). Autophagy is an intracellular degradation system that delivers cytoplasmic materials to the lysosome via the double-membraned autophagosome, which is highly conserved in the evolution of eukaryotes and includes the following steps: 1) autophagy initiation (signals activating autophagy) and nucleation of the phagophore/isolation membrane (IM); 2) phagophore elongation; 3) closure to form the autophagosome; 4) fusion between autophagosome and lysosome; 5) degradation of substrates in autolysosomes (Dikic and Elazar, 2018) (Fig. 1). Extensive studies have focused on the molecular mechanisms behind these processes, and our knowledge of them is constantly updated with discoveries. So far, there are many works reviewing autophagy from various angles. In the past four years, there are review papers covering topics including functions of autophagy in disease (Dikic and Elazar, 2018; Thorburn, 2018; Levine and Kroemer, 2019), selective autophagy (Gatica et al., 2018; Johansen and Lamark, 2020), transcriptional/post-transcriptional regulation in autophagy (Delorme-Axford and Klionsky, 2018), interaction network and structure of autophagic proteins (Behrends et al., 2010; Hurley and Young, 2017; Suzuki et al., 2017; Lai et al., 2019), molecular mechanisms of autophagy (Hollenstein and Kraft, 2020; Melia et al., 2020; Nakatogawa, 2020), and specific steps of autophagy, including autophagosome formation (Carlsson and Simonsen, 2015; Dikic and Elazar, 2018; Mercer et al., 2018; Otomo et al., 2018; Yu et al., 2018; Ktistakis, 2019; Lai et al., 2019; Osawa et al., 2019a; Osawa and Noda, 2019; Otomo and Maeda, 2019; Graef, 2020; Melia et al., 2020; Nishimura and Tooze, 2020), maturation/fusion (Nakamura and Yoshimori, 2017; Reggiori and Ungermann, 2017; Kriegenburg et al., 2018; Yu et al., 2018; Kriegenburg et al., 2019; Zhao and Zhang, 2019), and recycling (Yu et al., 2018). Most of the steps in autophagy are associated with membranes and are tightly regulated by membrane-associated proteins (Lystad and Simonsen, 2016; Shatz et al., 2016), so here we review autophagic lipids, membrane-associated autophagic proteins, and biochemical/biophysical assays applied in this field. We will emphasize three important membrane remodeling processes in autophagy, including lipid transfer for phagophore elongation, membrane scission for phagophore closure, and autophagosome-lysosome membrane fusion. We will highlight the discoveries in these processes, which provide new information on long-standing and important questions about autophagy and might direct future studies in this field.

## MEMBRANE COMPOSITION OF AUTOPHAGY-RELATED ORGANELLES

Each step in the autophagy pathway is defined by its membranes; membranes in turn are defined by their lipid and protein components, with PIs (phosphatidylinositols) and Rab GTPases (guanosine triphosphates) providing the main molecular determinants of organelle membrane identity in cells. The different lipid types combined with particular ratios and leaflet-specific asymmetrical distribution can give rise to particular membrane properties such as fluidity, curvature, and electrostatics. The lipid composition of autophagic organelles has been grossly identified (Table 1), but is not fully addressed, which is partly due to the difficulty of purifying different autophagic organelles. The lysosome is the only autophagic organelle that can be readily purified, and the lysosomes in rat liver contain 39% PC (phosphatidylcholines), 14% PE (phosphatidylethanolamine), 5% PI, 2% PS (phosphatidylserine), 1% cardiolipin, 1% PA (phosphatidic), and 20% sphingomyelin (Wherrett and Huterer, 1972; de la Ballina et al., 2020). Recently, the lipid composition of Atg8 membranes in yeast, which might represent yeast phagophore, autophagosome, and autolysosome, was reported to contain 38% PC, 37% PI, 19% PE, 3% PS, 3% PA, and are rich in unsaturation phospholipids. This is in agreement with the dynamic property of the phagophore (Schütter et al., 2020). Besides, compared to other cellular organelles, Atg8 (autophagy-related protein 8) membranes have a higher relative PI composition, which might be the reason for its important functions in autophagy. The formation of and transitions between these membranes are regulated by a series of membrane-associated proteins (Table 2), the full roles of which are still coming into focus. One important question that has been explored regarding the lipid components of autophagy is the membrane sources of the autophagosome.

**Table 1:** Lipid composition of autophagy membrane and other related subcellular fractions

	ER		Mitochondria		Plasma membrane		Vacuole/ Lysosomes		Atg8 membrane	Golgi
	Yeast	Mammalian	Yeast	Mammalian	Yeast	Mammalian	Yeast	Mammalian	Yeast	Mammalian
Phospholipid (mg/mg protein)	0.22	0.374	0.09	0.175	0.23	0.672	0.51	0.156		0.825
Sterols (mg/mg protein)	0.05	0.014	0.01	0.003	0.40	0.128	0.05	0.038		0.038
Sphingolipids (mg/mg protein)	0.14		0.02		0.27		0.20			
	% of total phospholipids									
PC	50	60	40	44	18	40	51	39	38	51
PE	24	23	27	34	22	24	21	14	19	21
PI	12	10	15	5	19	8	20	5	37	12
PS	9	2	3	<1	36	9	4	2	3	6
PA	4	1	2	ND	4	1	2	1	3	<1
Sphingomyelin		3		1		17		20		8
Cardiolipin	1	1	13	14	<1	1	2	1		1
References	(Zinser and Daum, 1995; Daum and Vance, 1997)	(Zambrano et al., 1975)	(Zinser and Daum, 1995)	(de Kroon et al., 1997)	(Zinser and Daum, 1995)	(Zambrano et al., 1975)	(Zinser and Daum, 1995)	(Wherrett and Huterer, 1972)	(Schütter et al., 2020)	(Zambrano et al., 1975)

Note: Mammalian quantification data comes from rat liver; All the lipids mentioned here are phospholipids, not lysophospholipids; ND stand for not detectable, and lipid composition with no available information is left blank.

**Table 2:** Direct membrane binding proteins in autophagy

Mammalian protein recruited	Yeast ortholog/ Function counterpart	Protein function in whole process	Membrane interaction ways	Spatiotemporal dynamic distribution					References
				I/N	E	S	M	AF	
ULK1/2	Atg1	Ser/Thr kinase in ULK1 complex, phosphorylating autophagy initiation machinery	C-terminal EAT domain	√					(Chan et al., 2009; Ragusa et al., 2012)
FIP200 (RB1CC1)	Atg11/ Atg17	Subunit of ULK1 complex (scaffold for autophagosome biogenesis)	α-helical structure	√					(Ragusa et al., 2012)
ATG13	Atg13	Subunit of ULK1 complex, regulating kinase activity of ULK1	Positively charged amino acids	√					(Karanasios et al., 2013)
ATG14, (ATG14L, Barkor)	Atg14	Subunit of PI3KC3C1 complex, anchoring complex to high curvature membrane; Tethering autophagosome and lysosome	C-terminal BATS domain	√			√		(Fan et al., 2011; Diao et al., 2015)
BECN1	Vps30 (Atg6)	Subunit of PI3KC3C1 complex, connecting complex to membrane;	C-terminal aromatic finger in BARA (beta-alpha repeated, autophagy-specific domain)	√			√		(Liang et al., 2008; Chang et al., 2019)
VPS15 (PIK3R4, p150)	Vps15	Subunit of PI3KC3C1 complex; Scaffold of the complex	Myristoylated N-terminus	√			√		(Slessareva et al., 2006)
PIK3C3 (VPS34)	Vps34	Phospholipid kinase in PI3KC3C1 complex	Kinase domain for PI binding; C terminal domain for membrane anchoring	√					(Petiot et al., 2000; Sun et al., 2011; Baskaran et al., 2012; Ma et al., 2017)
UVRAG	Vps38	Enhance autophagy induction and promote autophagosome-lysosome fusion	Phospholipid binding C2 domain	√			√		(Liang et al., 2008; He et al., 2013; Zhang et al., 2019)
WIPI2 WIPI4	Atg18 Atg21	PI3P binding protein: WIPI2 recruits ATG12~ATG5-ATG16L1 to phagophore, retrieves of ATG9 from early autophagosome membranes; WIPI4 can bind with ATG2 in the regulation of lipid transfer	6CD loop in ß-propeller structure	√	√				(Watanabe et al., 2012)
ATG2A ATG2B	Atg2	Transport lipids	N-terminal hydrophobic cavity and CAD tip can bind with PI3P interacting protein, WIPI4	√	√				(Watanabe et al., 2012; Osawa et al., 2019b; c; Valverde et al., 2019)
GRAMD1A	Lam1-6		Transmembrane region, GRAM domain, VASt domain		√				(Tong et al., 2018; Laraia et al., 2019)
VPS13A	Vps13a		‘Chorein_N’ domain		√				(Muñoz-Braceras et al., 2015)
ATG9A	Atg9	Delivery of membrane materials to the phagophore	Transmembrane domain	√	√				(Reggiori et al., 2005; Yamamoto et al., 2012)
SAR1A	Sar1p	GTP/GDP triggered switch to bind with ER membrane, further recruit Sec13p/31p complex, and archer COPII vesicles on ER membrane	N-terminal amphipathic a-helix	√	√				(Bielli et al., 2005; Omari et al., 2018)
Bif1	-	Membrane sculpting and protein scaffolding	N-BAR domain		√				(Takahashi et al., 2007; Takahashi et al., 2011)
SNX18	-		PX-BAR superdomain		√				(Knaevelsrud et al., 2013; Soreng et al., 2018)
ATG3	Atg3	Conjugate PE to ATG8 family	N-terminal amphipathic helix	√					(Ichimura et al., 2000; Nath et al., 2014)
ATG5	Atg5		Positively charged amino acids	√	√		√		(Chen et al., 2012; Romanov et al., 2012)
ATG16L1	Atg16		N-terminal membrane-binding amphipathic helix, CCD domain, and C-terminal β-isoform-specific membrane-binding region	√	√				(Matsushita et al., 2007; Fujioka et al., 2010; Dudley et al., 2019; Lystad et al., 2019)
ATG4	Atg4	processing Atg8 precursors and deconjugating Atg8-PE	Binding with lipidated LC3 via its LIR(LC3-interacting region) domain in mammalian or AIM(Atg8-interacting motif) domain in yeast and enzyme body	√	√		√		(Yu et al., 2012; Maruyama and Noda, 2018)
LC3 family proteins (LC3A, LC3B, LC3C, GABARAP, GABARAPL1, GABARAPL2)	Atg8	GABARAP: Phagophore formation; ULK activation; Fusion LC3: cargo docking; transport of autophagosome	PE-conjugation	√	√	√	√	√	(Kirisako et al., 1999; Kabeya et al., 2000; Johansen and Lamark, 2020)
DFCP1	-	As a PI3P effector, its function is unclear	FYVE domain	√	√				(Axe et al., 2008)
CHMP6	Vps20	Closure of phagophore	N-terminal myristylation			√			(Yorikawa et al., 2005)
CHMP4B	Vps32		N-terminal amphipathic helix			√			(Buchkovich et al., 2013; Zhen et al., 2019; Zhou et al., 2019)
CHMP3	Vps24		Positively charged residues; N-terminal amphipathic helix			√			(Buchkovich et al., 2013; Takahashi et al., 2018; Zhen et al., 2019)
CHMP2A	Vps2		Positively charged residues; N-terminal amphipathic helix			√			(Buchkovich et al., 2013; Takahashi et al., 2018; Takahashi et al., 2019)
VPS37A	Vps37		Positively charged N-terminal residues			√			(Kostelansky et al., 2007; Takahashi et al., 2019)
STX17	-	Fusion SNARE, promoting fusion between autophagosome and lysosome	Transmembrane domain				√		(Itakura et al., 2012; Jiang et al., 2014; Diao et al., 2015)
YKT6	Ykt6		N-terminal longin domain				√		(Matsui et al., 2018)
VAMP8	-		Transmembrane domain				√		(Itakura et al., 2012; Diao et al., 2015)
STX7	-		Transmembrane domain				√		(Matsui et al., 2018)
HOPS complex (VPS11, VPS16, VPS18, VPS33A, VPS39, VPS41)	HOPS complex (Vps11, Vps16, Vps18, Vps33, Vps39, Vps41)	Tethering between autophagosome and lysosome	By binding with Rabs, Rab effectors, SNAREs, membrane lipids and etc.				√		(Stroupe et al., 2006; Jiang et al., 2014)
RAB2	Ypt2		C-terminal prenylation				√		(Ding et al., 2019)
RAB7	Ypt7		C-terminal prenylation				√		(Carroll et al., 2013)
TECPR1	Pex23p		Pleckstrin homology (PH) domain				√	√	(Jeynov et al., 2006; Ogawa et al., 2011; Chen et al., 2012; Xu et al., 2015; Wetzel et al., 2020)
αSNAP	Sec17	Disassemble SNARE complex with NSF	Hydrophobic amino acids and SNAREs				√	√	(Zhao et al., 2015; White et al., 2018)

I/N-Initiation/Nucleation; E-Elongation; S-Scission; M-Maturation; AF-After fusion

It is generally agreed upon that phagophore is formed *de novo* by nucleation on the ER (endoplasmic reticulum) membrane compartment (Shibutani and Yoshimori, 2014). Recently Martin Graef and his colleagues proposed a new perspective on autophagosome biogenesis (Schütter et al., 2020). They found that localized *de novo* synthesized phospholipids from the ER are likely the main membrane sources for phagophore expansion rather than lipids from preformed organelle membranes. They proposed this might help to maintain the integrity of existing organelles. Besides, they found the Acyl-CoA synthetase Faa1 (long-chain-fatty-acid-CoA ligase 1) is recruited to PAS (pre-autophagosomal structure) upon autophagy initiation to activate fatty acids for further synthesis of phospholipids on the ER, but it remains unclear how Faa1 is recruited from the ER to the phagophore to activate the first step of phospholipid synthesis. It’s generally accepted there are three possible ways to transfer lipids for phagophore membrane extension: vesicle-mediated delivery, lipid transfer protein-mediated delivery (e.g., ATG2, autophagy-related gene 2), and maybe direct extrusion from pre-existing organelles, all of which we are going to discuss in the second part of this review paper.

Another outstanding question concerns the function of different PIs. Among all the phospholipids, PIs are signaling molecules that play important roles in autophagy, as they can confer unique membrane identity and recruit particular protein machinery with high spatiotemporal control. The inositol ring of phosphatidylinositol can be phosphorylated on three, four, and five hydroxyl groups in different combinations to generate seven distinct PIs. There are works specifically reviewing the roles of phosphoinositides in autophagy in detail (Jang and Lee, 2016; Chung, 2019; Palamiuc et al., 2020). During the biogenesis of the autophagosome, PI3P (phosphatidylinositol-3-phosphate) can be generated from PI on the omegasome, phagophore and contact sites by the PI3KC3C1 complex (class III phosphatidylinositol-3-kinase complex I) (Schu et al., 1993; Russell et al., 2013). PI3P recruits the ATG16L1-ATG5~ATG12 complex through WIPI2 (WD repeat domain phosphoinositide-interacting protein 2) for conjugating PE to LC3 (microtubule-associated proteins 1A/1B light chain 3) (Hanada et al., 2007; Fujita et al., 2008). PI4P (phosphatidylinositol-4-phosphate) and PI5P may act in an alternative function for PI3P when binding WIPI2 for autophagosome biogenesis. During phagophore elongation, PI3P recruits ATG2 via WIPI4 to transfer lipids for phagophore expansion (Osawa et al., 2019c; Tang et al., 2019; Valverde et al., 2019). PI4P also mediates the exit of ATG9 vesicles from the Golgi complex to the phagophore and promotes the expansion of phagophore (Mizushima et al., 2011; Wang et al., 2012). Besides, both PI(3,5)P2 (phosphatidylinositol-3,5-phosphate) and PI(4,5)P2 (phosphatidylinositol-4,5-phosphate) can recruit machinery for the expansion of phagophore (Ho et al., 2012; Wang et al., 2012; Carroll et al., 2013; Knaevelsrud et al., 2013), such as ATG16L1 and SNX18 (sorting nexin-18) (Ravikumar et al., 2010; Knaevelsrud et al., 2013; Soreng et al., 2018). During fusion, PI3P binds with TECPR1 (tectonin beta-propeller repeat-containing protein 1) on the lysosome and promotes autophagosome-lysosome fusion (Chen et al., 2012). On the other hand, PI3P on the autophagosome and PI4P on the late endosome/lysosome can recruit HOPS (homotypic fusion and protein sorting complex) to facilitate autophagosome maturation (Bas et al., 2018) and promote the trafficking of lysosome proteins to increase the degradative capability of the lysosome (Miao et al., 2020). The conversion between PI3P to PI(3,5)P2 and PI4P to PI(4,5)P2 are both important for fusion events (Hasegawa et al., 2016; Li and Zhong, 2016; Baba et al., 2019). After fusion, the conversion from PI4P to PI(4,5)P2 is necessary for the autophagic lysosome reformation from the autolysosome, where PI(4,5)P2 can recruit clathrin to autolysosomes and may control the fission of reformed tubules from autolysosomes (Rong et al., 2012). Besides, the level of PI(3,5)P2 is a marker of mature autophagosomes and may be required for membrane recycling after fusion with lysosomes (Dove et al., 2009).

Altogether, the lipid composition determines the property of autophagic organelles and plays a critical function in regulating autophagy. However, there are still many open questions that need to be answered. For example, VPS34 (vacuolar protein sorting 34) is one source of PI3P, but PI3P can also be generated by Class II PI 3-kinases and phosphatases (Nascimbeni et al., 2017). Whether the VPS34-independent source of PI3P is important for autophagy is not fully understood. A better understanding of the source of PI3P can complete the model of the regulation of autophagy and may give us new targets for drug discovery. Besides, it will be helpful to complete the PI regulatory network in the future for fully understanding the autophagy pathway, including the autophagic binding proteins, the balance of PI abundance, and their correlation with disease.

## MEMBRANE-ASSOCIATED PROTEINS

Autophagy is a membrane-driven process and is spatiotemporally regulated by a series of membrane-associated proteins (Fig. 1). The initiation of autophagy starts when cells sense stimulation signals. The omegasome (a PI3P-rich subdomain of ER, marked by DFCP1 (double zinc-finger FYVE domain-containing protein 1)) is the platform for phagophore formation. This step involves two important complexes: the ULK1 (autophagy activating kinase 1)/Atg1 complex (composed of ULK1/2, ATG13, ATG101, and FIP200 (focal adhesion kinase family interacting protein of 200 kDa) in mammalian cells and Atg1, Atg13, Atg17, Atg29, and Atg31 in yeast) and the PI3KC3C1 complex (composed of Beclin1, ATG14, VPS34, VPS15, NRBF2 in mammalian cells and Vps30, Atg14, Vps34, Vps15, Atg38 in yeast) (Russell et al., 2013). It was recently found that the yeast Atg1 complex might undergo phase separation for self-activation and the liquidation of PAS. This would facilitate the incorporation of Atg9 vesicles, one of the initial sources of autophagosomal membranes, possibly by heterotypic fusion of vesicles (Hosokawa et al., 2009; Jung et al., 2009; Ragusa et al., 2012; Karanasios et al., 2013; Fujioka et al., 2020). The activated ULK1/Atg1 kinase complex triggers the nucleation of phagophore by phosphorylating components of the PI3KC3C1 complex (Sun et al., 2008; Fan et al., 2011; Ragusa et al., 2012; Russell et al., 2013). Once activated, the PI3KC3C1 complex can generate more PI3P on the phagophore, which then recruits autophagic core machinery through WIPIs (WD repeat domain phosphoinositide-interacting proteins) (Petiot et al., 2000; Sun et al., 2011; Baskaran et al., 2012; Ma et al., 2017), such as ATG12~ATG5-ATG16L1 via WIPI2 (Kabeya et al., 2000; Nakatogawa et al., 2007; Pankiv et al., 2007; Xie et al., 2008; Polson et al., 2010) and ATG2 via WIPI4 (Chowdhury et al., 2018; Maeda et al., 2019; Osawa et al., 2019b). Once the phagophore is nucleated, it starts to expand to engulf cargo substrates. The LC3 is then conjugated with PE to decorate the phagophore membrane and plays a role in the recognition of substrates in selective autophagy. The conjugation systems in autophagy were extensively reviewed (Johansen and Lamark, 2020). During membrane elongation, there are three possible sources of membranes that will be discussed in the next section (Mizushima, 2007). Then, the sealing of this phagophore membrane structure by scission proteins ESCRT (endosomal sorting complexes required for transport) and other machinery gives rise to a double-layer autophagosome (Takahashi et al., 2018; Takahashi et al., 2019; Zhen et al., 2019; Zhou et al., 2019). Autophagosomes and lysosomes are tethered by different tethering machinery, including Rab GTPases, effector proteins of Rabs (e.g., HOPS), ATG14, etc. (Jiang et al., 2014; Diao et al., 2015; Ding et al., 2019). So far the signal for triggering autophagosome-lysosome fusion is still unknown, but it is thought to be driven by SNAREs (soluble NSF attachment protein receptors), including STX17 (syntaxin 17)-SNAP29 (synaptosomal-associated protein 29)-VAMP8 (vesicle- associated membrane protein 8), and STX7-SNAP29-YKT6 (Itakura et al., 2012; Diao et al., 2015; Matsui et al., 2018). Once fused, acidic hydrolases in the lysosome can degrade the autophagic cargos and salvaged nutrients are released to the cytoplasm to be recycled by cells (Rong et al., 2012; Yu et al., 2018; Zhao and Zhang, 2019). The detailed molecular mechanisms of these proteins regulating different steps in autophagy have been recently reviewed in separate papers (Carlsson and Simonsen, 2015; Nakamura and Yoshimori, 2017; Reggiori and Ungermann, 2017; Dikic and Elazar, 2018; Mercer et al., 2018; Yu et al., 2018; Lai et al., 2019; Osawa et al., 2019a; Zhao and Zhang, 2019; Melia et al., 2020; Nishimura and Tooze, 2020). In Table 2, we summarized all of the known membrane-associated proteins, their functions, and their membrane-binding mechanisms in all steps of autophagy in a spatiotemporal way. The proteins which are not yet reported to directly associate with membrane are not listed. Viewing these membrane-associated autophagic proteins in a spatiotemporal way can help us to have a full insight of autophagy mechanism, and understand the dynamic of proteins and lipids in autophagy, like when and where a lipid or protein is recruited and when it leaves the membrane. For example, STX17 is only recruited to closed autophagosomes (Itakura et al., 2012), and its co-localization with LC3 can be used as an indicator for mature autophagosome (Tsuboyama et al., 2016). Besides, the spatiotemporal dynamic distribution information can help us to find the possible sensors for autophagic membrane shaping/sculpting in future study. Furthermore, we will discuss the discoveries and research trends of three important membrane remodeling processes in the following section, including phagophore membrane elongation, phagophore membrane scission and autophagosome-lysosome membrane fusion. These discoveries shed a light on the long-standing questions in the autophagy field and might direct a research trend for future studies.

## PHAGOPHORE MEMBRANE ELONGATION

The phagophore membrane extends while engulfing substrates destined for degradation. Upon stimulation, macroautophagy probably needs to mobilize millions lipid molecules per cell for autophagosome growth (Melia et al., 2020). However, the molecular mechanism of how membrane elongation works is not fully understood. There are three possible ways to contribute to phagophore membrane elongation, including vesicle-mediated delivery, direct extrusion from pre-existing organelles, and lipid transfer protein-mediated direct delivery (Melia et al., 2020).

For vesicle-mediated delivery, ATG9-containing vesicles formed from recycling endosomes and the Golgi apparatus (dependent on SNX18 and other proteins) (Reggiori et al., 2005; Takahashi et al., 2011; Yamamoto et al., 2012; Gómez-Sánchez et al., 2018; Mercer et al., 2018; Tang et al., 2019) and COPII vesicles from ERGIC (ER-Golgi intermediate compartment) have been implicated in phagophore elongation (Ishihara et al., 2001; Ge et al., 2013; Graef et al., 2013; Ge et al., 2014; Stadel et al., 2015). It was observed that during starvation, several Atg9 vesicles assembled individually into the pre-autophagosomal structure and were eventually incorporated into the autophagosomal outer membrane (Yamamoto et al., 2012); ERGIC-derived COPII vesicles functioning as a membrane template for LC3 lipidation are recruited to forming autophagosomes (Ge et al., 2013; Ge and Schekman, 2014; Ge et al., 2017) and become part of autophagosomal membranes (Shima et al., 2019). However, how these vesicles fused with pre-autophagosomal structures is still elusive. SNAREs are the core machinery driving membrane fusion in general and are required for autophagosome biogenesis (Moreau et al., 2011; Nair et al., 2011; Puri et al., 2013; Lemus et al., 2016), but so far there is no direct evidence to show that the fusion between the phagophore and ATG9 vesicles or COPII vesicles is SNARE-driven.

Some studies have observed the direct connections between the phagophore/IM and ER, which suggests the cup-shaped phagophore might directly be extruded from the ER (Hayashi-Nishino et al., 2009; Ylä-Anttila et al., 2009). Besides, some studies demonstrated an LC3-positive structure forming directly on extruded regions of the mitochondrial outer membrane, which suggest phagophore growth by extrusion from mitochondria (Hailey et al., 2010). However, this extrusion model is still under active debate and how transmembrane proteins are excluded during membrane utilization is unclear.

Another model is direct protein-mediated lipid transfer, where the lipid can be transferred directly by lipid transfer protein from the ER to the phagophore/IM. In the direct transfer model, no fusion machinery is needed. In addition, the influx of membrane proteins to the phagophore/IM can be excluded as the phagophore membrane and ER are not directly attached. Direct protein-mediated lipid transports for autophagosome extension are intensive studied recently. At least four lipid transfer proteins have been implicated in autophagosome biogenesis, including ATG2, GRAMD1A (GRAM domain-containing 1A), VPS13A, and TipC (putative vacuolar protein sorting-associated protein 13C) (Muñoz-Braceras et al., 2015).

**Figure 2:**
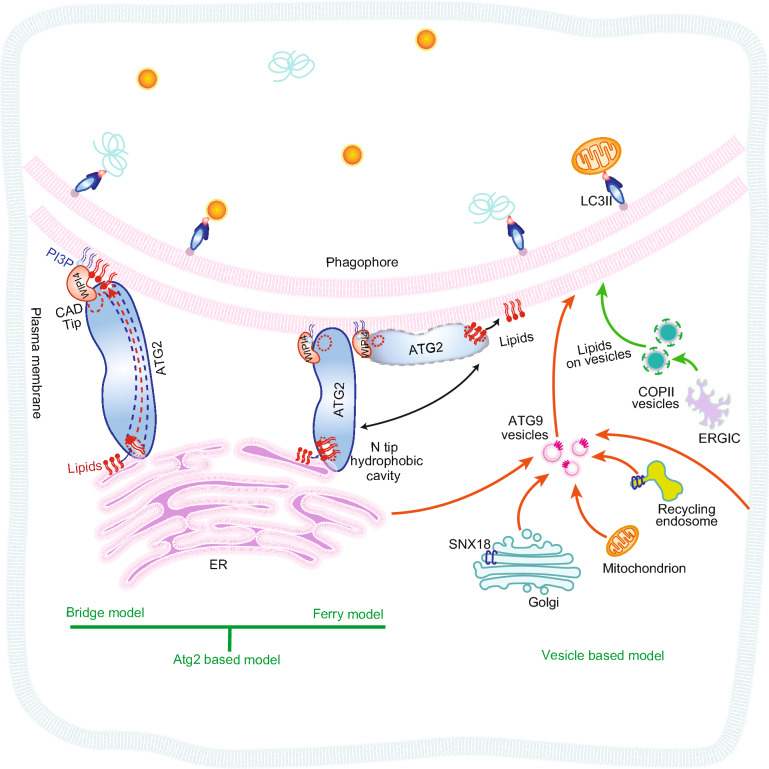
**Hypothetic model for ATG2-mediated lipid transfer during autophagy**. ATG2, ATG9-vesicles, and COPII-vesicles help to provide membrane sources for phagophore membrane elongation. ATG2 contains a CAD tip that binds to the PI3P interacting protein WIPI4 and an N-terminal hydrophobic cavity that binds to lipids. Inside the protein, there is an extended cavity or a series of cavities along the length of it. Two models are presented for the mechanism of lipid transfer for ATG2, the bridge-tunnel model (left), and the ferry model (middle). In the bridge model, ATG2 stably tethers the phagophore and ER, where its CAD tip interacts with WIPI4, a PI3P effector on the phagophore and its N tip hydrophobic cavity binds to the ER. The lipids could transfer in the tunnel inside ATG2 from the ER to the phagophore. In the ferry model, the CAD tip anchors on the phagophore membrane through WIPI4, while the N tip hydrophobic cavity takes lipids from ER, and swings like a ferry boat between the ER and the phagophore to transfer lipids. Simultaneously, ATG9-vesicles and COPII-vesicles can act in a vesicle-mediated membrane fusion to deliver lipids from many cellular organelles to the phagophore for its elongation

Atg2 was first identified alongside 12 other ATG (autophagy-related) genes from a screen of *S*. *cerevisiae* autophagy defective mutants by the Oshumi group in 1993 (Tsukada and Ohsumi, 1993). Its function for autophagosome formation in yeast and mammalian cells was established by Oshumi group in 2001 (Shintani et al., 2001) and Mizushima group in 2012 (Velikkakath et al., 2012), respectively. The lipid-binding and membrane-tethering (MT) activity of ATG2 was identified at earlier time, however, the lipid transfer (LT) activity of ATG2 was not characterized until recent studies. A series of *in vitro* biochemistry experiments demonstrated that both mammalian ATG2A and ATG2B can extract and unload lipids and transfer lipids between tethered membranes *in vitro*, which suggests that ATG2 can mediate lipid transfer between the ER and the phagophore (Maeda et al., 2019; Osawa et al., 2019b, c; Valverde et al., 2019). Both human and yeast ATG2 have an N-terminal Chorein domain, and an X-ray structure of the N-terminal region (NR) of yeast Atg2 co-crystallized with PE showed the acyl chain of the phospholipid can be buried inside the hydrophobic cavity of the N-terminal region. Disruption of the interaction between PE and the hydrophobic cavity by mutagenesis causes defects in phagophore/IM elongation *in vivo* and in MT and LT activity *in vitro* (Osawa et al., 2019c). The reconstituted structure of mammalian ATG2A by 3D cryo-EM (cryogenic electron microscopy) showed an extended cavity or a series of cavities along the length of ATG2A, which might be a tunnel for the lipid to be transferred inside (Valverde et al., 2019). Besides, the Otomo group used negative staining and CXL-MS to identify the interaction between the CAD (cysteine-alanine-aspartic acid triad) tip of ATG2A and blade 2 of WIPI4, whose blades 5 and 6 bind to PI3P on the phagophore (Chowdhury et al., 2018) and their lipid transfer assays showed that ATG2A can transfer lipids between SUVs (small unilamellar vesicles) without WIPI4. However, lipid transfer between LUVs (large unilamellar vesicles) by ATG2A is dependent on WIPI4 (Maeda et al., 2019). So far, there are two possible lipid transfer models of ATG2 that are generally accepted, one is the bridge model, and the other is the ferry model (Fig. 2) (Maeda et al., 2019). In both models, the CAD tip of ATG2 binds the phagophore through interacting with the PI3P effector WIPI4. In the bridge model, ATG2 stably tethers the phagophore to the ER membrane. With its N-terminal region anchored on the ER membrane, ATG2 takes lipids from the ER membrane and transfers them through its hydrophobic tunnel. In the ferry model, the N terminus of ATG2 is not stably anchored on the ER membrane, instead, it dynamically swings between the ER and phagophore without transferred lipids through the hydrophobic tunnel. These theories inspire new possible mechanisms for phagophore expansion, which can help us to solve the puzzle of autophagy biogenesis, but many questions remain to be answered. First, the cell need transfer millions lipids in ~10 min to complete one autophagosome (Melia et al., 2020). The measured lipid transfer rate of ATG2 *in vitro* is about 0.017 lipid/ATG2 molecular/sec (Maeda et al., 2019). It remains unknown whether high local concentration of ATG2, ATG2 conformational change and binding partners are required to achieve biologically meaningful lipid transfer rate to meet the need of autophagosome growth. Besides, it was observed that lipid transfer by ATG2 is bi-directional *in vitro*, which is different from the expectation that the lipid transfer should be unidirectional from ER to phagophore *in vivo*. These results suggest there are other proteins or factors functioning together with ATG2 to maintain efficient lipid transfer and provide the energy source for unidirectional transport. Besides, what regulators drive lipid preference? An interaction-based protein screening assay might provide a way to look for possible regulators of ATG2. Furthermore, what is the lipid transfer model for ATG2: bridge model, ferry model, or another? Previously, it was observed that the N-terminal 1–345 amino acid fragment of ATG2A is enough to rescue autophagosome formation deficiency in ATG2A/B double knockout cells, which suggests ATG2A can function without its C-terminal domain. Finally, how does ATG2 cooperate with ATG9 vesicle to transfer lipids? New tools for visualizing such processes might be required to monitor lipid transfer in live cells.

GRAMD1A (GRAM domain-containing 1A), also known as Aster-A, is a cholesterol transfer protein (biochemical and structure evidence) in the StART (steroidogenic acute regulatory protein-related lipid transfer) domain family (Besprozvannaya et al., 2018; Naito et al., 2019). It contains a transmembrane region, a phosphoinositide-binding GRAM domain, and a cholesterol-binding VASt domain. Upon starvation, GRAMD1A accumulates at autophagosome initiation site possibly through PI3P binding. The selective inhibition of GRAMD1A or siRNA knockdown of GRAMD1A inhibits autophagosome biogenesis (Laraia et al., 2019). However, there is no direct evidence to show that this protein transfers cholesterol for phagophore membrane extension. Also, it is not clear whether GRAMD1B and GRAMD1C have a similar function in autophagy.

VPS13 was identified as lipid transfer protein with a “Chorein_N” domain at the N-terminus and an “ATG_C” domain at the C-terminus (autophagy-related protein C-terminal domain), similar to Atg2 (Kumar et al., 2018). VPS13 proteins localize to multiple membrane-contact sites. Human VPS13A/VPS13C localizes on ER, tethering ER to mitochondria (VPS13A), to late endosome/lysosomes (VPS13C), and lipid droplets (both VPS13A and VPS13C) (Kumar et al., 2018). The tethering specificity was determined by adaptor proteins from different organelles. It was found that Ypt35, Spo71, and Mcp1 can compete to recruit Vps13 to endosomes/vacuoles/NVJ (nucleus-vacuole junction), the prospore membrane, and mitochondria, respectively, in yeast (Bean et al., 2018). VPS13A was reported to regulate autophagy in human HeLa cells. Downregulation of VPS13A in HeLa cells caused the accumulation of autophagic markers and impaired autophagic flux (Muñoz-Braceras et al., 2015). Besides, TipC from Dictyostelium, highly similar to the VPS13 family of proteins, is also reported to regulate autophagy in Dictyostelium. Dictyostelium cells lacking TipC displayed a reduced number of autophagosomes and impaired autophagic degradation. However, so far there is no evidence to show that TipC can transfer lipids *in vitro*, even though it’s highly homology with human VPS13A and VPS13C. Besides, there is no direct evidence to show TipC function in phagophore membrane elongation.

Taken together, more studies are needed to fully characterize these lipid-transfer models, including the lipid preference by key players within these models, and how these lipid-transfer machinery coordinate with each other to complete the phagophore membrane extension.

## ESCRT-MEDIATED MEMBRANE SCISSION FOR AUTOPHAGOSOME COMPLETION

According to the membrane topology, the phagophore with one membrane must go through membrane scission to generate the autophagosome with a double membrane. This may occur via ESCRT, however, its function in phagophore closure was not established until recently (Takahashi et al., 2018; Takahashi et al., 2019; Feng et al., 2020). The ESCRT complexes are groups of highly evolutionarily conserved membrane remodeling machinery that act in many cellular membrane scission processes, like cytokinetic abscission, plasma membrane vesicle budding, and endosomal sorting. 15 of the ESCRT proteins were first identified as a subgroup of VPS (vacuolar protein sorting) genes in yeast that are required for protein sorting (Raymond et al., 1992; Odorizzi et al., 1998). Together with other ESCRT proteins found later, they can be classified into ESCRT-0, ESCRT-I, ESCRT-II, ESCRT-III, and ESCRT-associated proteins (Komada and Kitamura, 1995; Asao et al., 1997; Katzmann et al., 2001; Babst et al., 2002a; Babst et al., 2002b). Based on a previous study *in vitro* using yeast proteins, it is generally accepted that ESCRT-I, ESCRT-II, ESCRT-III, and ESCRT-associated proteins constitute the core of ESCRT machinery (Schoneberg et al., 2017). ESCRT-I and ALIX (ALG2-interacting protein X) form a super complex to act as specific regulators to recruit ESCRT-III proteins to their scission site. ESCRT-I and ESCRT-II co-localize at the necks of membrane buds and recruit ESCRT-III to cleave the buds to form an intraluminal vesicle (Wollert and Hurley, 2010). ESCRT-III is also important for remodeling membranes as it can form membrane-interacting oligomeric filaments, flat spirals, tubes, and conical funnels to remodel membrane shape, and eventually scissor the membrane (Schoneberg et al., 2018). The N-terminal residues of ESCRT-III proteins are positively charged and provide the membrane-binding ability, while the other residues of these proteins are negatively charged. It is thought that the N-terminal parts provide the filamenting ability while the C-terminal helices regulate its activity (Schoneberg et al., 2018). After scission, the AAA+ ATPase VPS4 forms a hexamer to disassemble and recycle the ESCRT-III complex (Caillat et al., 2015; Schoneberg et al., 2018). The function and mechanism of ESCRT-III has been intensively studied, but the detailed mechanisms of scission are not fully understood (Schoneberg et al., 2018; Gatta and Carlton, 2019).

**Figure 3:**
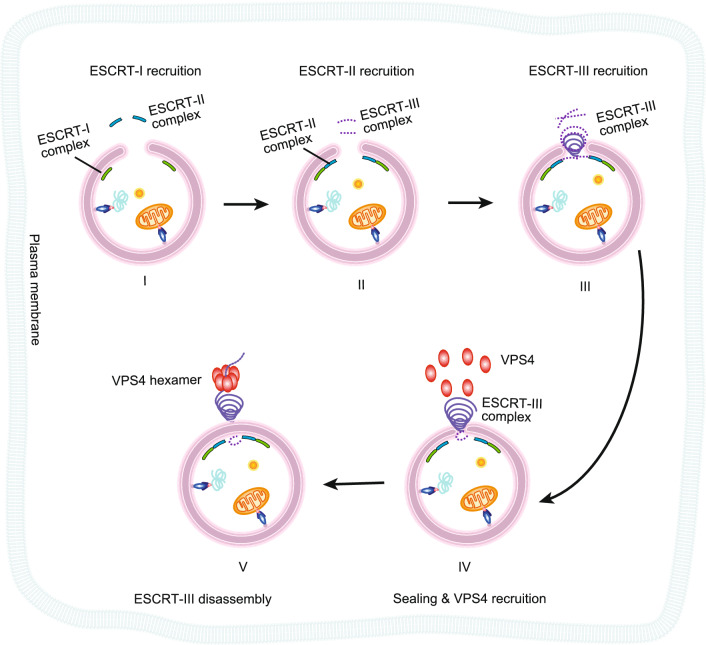
**Hypothetic model for ESCRT-mediated autophagosome pore closure**. Phagophore pore closure model was plotted according to the ESCRT-mediated membrane scission, as the pore closure might share the same regulators and mechanism with the classic ESCRT model. The first step of phagophore closure is the recruitment of the ESCRT-I complex and other related machinery to the phagophore pore. ESCRT-I recruits the ESCRT-II and ESCRT-III complex. ESCRT-III will form oligomeric filaments, flat spirals, tubes, and conical funnels to remodel the membrane shape on the neck, and finally, scissor the membrane to form a closed double-membrane autophagosome. After scission, the AAA+ ATPase VPS4 is recruited and forms a hexamer to disassemble and recycle the ESCRT-III complex

Recent studies on ESCRT provided some important clues about its function on the phagophore to form a closed double-membrane autophagosome (Takahashi et al., 2018; Zhen et al., 2019; Zhou et al., 2019). Y. Takahashi et al. developed a HaloTag-based autophagy assay to monitor the closure of the autophagosome (Takahashi et al., 2018; Takahashi et al., 2019) and found that CHMP2A (charged multivesicular body protein 2A), VPS4, and VPS37A are critical for this process. The ESCRT-I complex component VPS37A can associate with the phagophore by its N-terminal domain, which is critical for autophagosome completion, but not for endocytosis. Further studies found that VPS37A can recruit the ESCRT-I subunit VPS28 and the ESCRT-III subunit CHMP2A to the phagophore (Takahashi et al., 2019). Besides, using live-cell imaging Yan Zhen et al. demonstrated that the ESCRT-III component CHMP4B is recruited to an unsealed autophagosome, and the depletion of the ESCRT-III disassembly regulator VPS4 results in the accumulation of CHMP4 on autophagosomes for both canonical and selective autophagy (Zhen et al., 2019). More interestingly, another study on neurons shows that in neurodegenerative diseases, MAPT/Tau (microtubule-associated protein tau) accumulation represses autophagy flux by disrupting IST1 (increased sodium tolerance 1)-regulated ESCRT-III complex formation (Feng et al., 2020). Over-expression of IST1 in mice reduced the level of MAPT aggregation and ameliorated synaptic plasticity and cognitive functions. The relationship between autophagy and neurodegenerative disease has long been a mystery, especially concerning the clearance of TAU protein aggregation from neuron cells. It is believed that autophagy can be utilized to clear TAU protein aggregates. ESCRT-III might be a link between autophagy and MAPT/TAU in neurodegenerative diseases. Besides, a study in yeast shows that RAB5 recruits Atg17 (FIP200 in mammals) to the nascent autophagosome, and facilitates interactions between Atg17 and the ESCRT-III component Snf7 (CHMP4 in mammals) (Zhou et al., 2019). According to the recent discoveries in autophagy and combined with the general membrane scission mechanism established in other pathways, we proposed a possible model for how ESCRT complexes function in autophagosome closure (Fig. 3). All these studies shed a light on how phagophores are sealed, indicating that ESCRT complexes play a role in autophagy, most likely at scission steps. The self-labeling tags and the membrane-permeable/impermeable dyes used here are the key points for these studies and have been summarized in a previous review (Rusten and Stenmark, 2009), there are controversies about the function of ESCRT in autophagy in yeast and other organisms. For example, in a previous study, deletion or functional loss mutantion of VPS4 caused the accumulation of autophagosomes, but not unsealed autophagosomes in yeast (Preiss, 2017). The different results might be explained by the functional differences in the Vps proteins. In another example, loss of Vps27, Snf7, or Vps4 increased lipid droplet turnover in *S*. *cerevisiae* (Zhang et al., 2020), suggesting an inhibitory function of ESCRT complexes in lipophagy rather than acting as a positive regulator.

More studies are needed to answer these questions and to draw a full picture of ESCRT in autophagy. Unlike in endosome sorting, the biochemical activity of ESCRT in the autophagic membrane is still unknown. An *in vitro* reconstitution system needs to be established to fully address the function of ESCRT in autophagy. Other questions include, how is ESCRT machinery recruited at autophagy-specific scission steps, and how are they regulated? Are there any autophagy-specific regulators correlated with these autophagic scission steps? How are ESCRT complexes triggered to start scission on phagophore? A high-throughput screening assay targeting genes involved in autophagosome closure could provide a way to look for these regulators. Besides, does ESCRT-mediated scission also work in other steps of autophagy, such as the formation of isolation membranes, generation of ATG9 or COPII vesicles for delivering lipids, recycling of lysosomes after fusion, etc. (Schoneberg et al., 2017). Our knowledge of the molecular mechanisms of these processes is very limited, and more work needs to be done to answer these essential questions.

## AUTOPHAGOSOME-LYSOSOME FUSION

The autophagosome delivers engulfed substrates to the lysosome for degradation via membrane fusion. To accomplish this fusion, first, the double-membraned autophagosome is tethered to the single-membrane lysosome; then, the outer membrane of the autophagosome fuses with the lysosome membranes; and finally, the inner membrane of the autophagosome is hydrolyzed by lysozyme and the autophagosomal contents are degraded. The process of membrane fusion is highly conserved in evolution. It is widely accepted that membrane fusion in general is driven by the zippering of the SNARE complex to form a four-helix bundle. In mammalian cells, the first set of SNARE complexes identified for autophagosome-lysosome fusion was STX17-SNAP29-VAMP8 (Itakura et al., 2012). Structure biology study showed that this complex can form a four-helix bundle as the other SNAREs and *in vitro* biochemistry reconstitution showed that they can drive the fusion between two liposomes (Diao et al., 2015), which we are going to discuss more in the next section. A recent study showed that YKT6 can bind to autophagosomes through its N-terminal longin domain. Together with SNAP29 and lysosomal STX7, a YKT6-SNAP29-STX7 complex can drive fusion (Bas et al., 2018; Matsui et al., 2018). However, more studies are required to figure out the functional difference between these two SNARE complexes. Furthermore, it was found that ATPase NSF (N-ethylmaleimide sensitive factor) and αSNAP (soluble NSF attachment protein) are required for priming of autophagic SNAREs (Ishihara et al., 2001; Abada et al., 2017), possibly by forming a super complex with the cis-SNARE complex and using the energy released from ATP hydrolysis to disassemble SNAREs as for neuro SNAREs (Zhao et al., 2015; Huang et al., 2019). The details of this mechanism still need to be investigated through biochemical and structural analysis.

**Figure 4:**
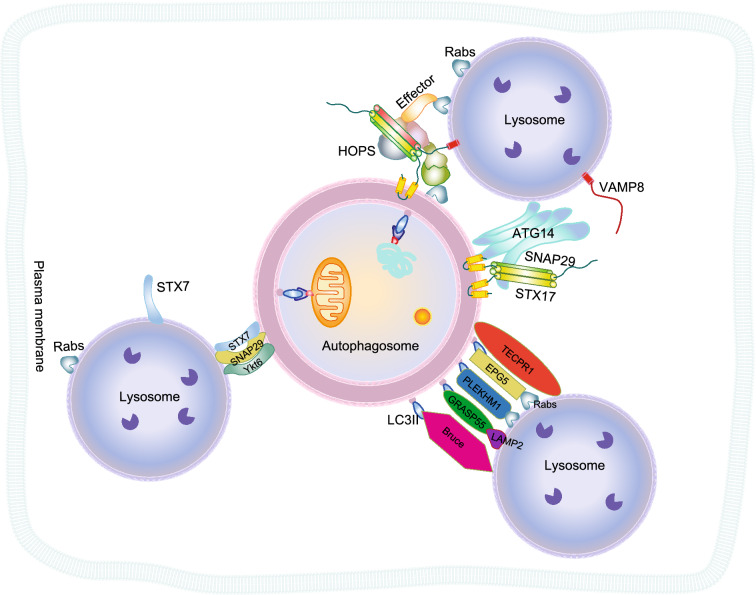
**Model for SNARE-mediated autophagosome-lysosome fusion**. Autophagosome-lysosome fusion is the key step of autophagy and is highly regulated by SNARE proteins, tethering factors, Rab GTPase, SM proteins, and other proteins. The fusion SNAREs identified so far for autophagy include STX17-SNAP29-VAMP8 and YKT6/SNAP29/STX7. Sealed autophagosome recruits the SNARE binary complex together with lysosomal SNARE protein to form a four-helix bundle to mediate autophagosome-lysosome fusion. Besides, tethering between autophagosomes and lysosomes can promote this fusion. Proteins involved in tethering include ATG14, Rab GTPase, HOPS, PLEKHM1, UVRAG, EPG5, BRUCE, TECPR1, GRASP55, etc

Beside SNAREs, other proteins are required to complete fusion efficiently (Fig. 4), including tethering proteins, Rab GTPases, SM proteins (Sec1/SM family proteins), and others (Wickner and Rizo, 2017). Qing Zhong and his colleagues demonstrated that cysteine-mediated oligomerization of ATG14 promotes the fusion of autophagosomes and lysosomes by tethering these two organelles and it can assist STX17 to recruit SNAP29 to autophagosomes promoting the assembly of SNARE complex (Diao et al., 2015). Besides, from the genetic analysis, the HOPS complex also plays a crucial role in the fusion between autophagosomes and lysosomes, likely through tethering of these two organelles together with SNAREs, Rabs, and other tethering related proteins such as PLEKHM1 (Pleckstrin homology domain-containing family M member 1) (McEwan et al., 2015), UVRAG (UV radiation resistance-associated protein) (Liang et al., 2008), EPG5 (Ectopic P granules protein 5 homolog) (Wang et al., 2016), BRUCE (BIR repeat-containing ubiquitin-conjugating enzyme) (Ebner et al., 2018), TECPR1 (Chen et al., 2012), GRASP55 (Golgi reassembly-stacking protein 55) (Zhang et al., 2019), and etc. Together, these proteins might behave as a template for SNAREs to register and assemble on it, or to expand the fusion pore as we learned from the biochemical studies of yeast vacuole fusion (Liang et al., 2008; Jiang et al., 2014; McEwan et al., 2015; Ding et al., 2019). However, the biochemical mechanism of HOPS in autophagy remains elusive. In yeast vacuolar fusion, the HOPS complex interacts with Ypt7 (the yeast homolog of human Rab7) to mediate vacuole fusion. Interestingly, in mammalian cells, HOPS does not directly interact with RAB7 (Ras-related protein Rab-7) (McEwan et al., 2015) and how Rabs mediate the membrane association of the HOPS complex to the autophagosome membrane and the lysosome membrane is enigmatic. Emerging evidence suggested that RAB2 (Ras-related protein Rab-2) might be the Rab GTPase mediating the interaction between HOPS and the autophagosome membrane (Ding et al., 2019). Other Rabs mediate the direct interaction between lysosomes and HOPS are yet to be identified. So far there are more than 60 human Rab proteins and 11 yeast Rab-related Ypt proteins reported. It is not completely understood how these Rab GTPases and their effectors discriminate among different trafficking pathways, which may converge at the same organelle but towards different goals. For example, how does the tethering effector HOPS complex on lysosomes differentiate fusion targets among autophagosomes, late endosomes, and AP3 (AP-3 complex subunit delta-1) vesicles from the Golgi? Furthermore, it is highly likely that HOPS and Rabs can coordinate with GEFs (guanine nucleotide exchange factors), other Rab effectors, and SNAREs to fulfill the complicated assignment of membrane tethering and fusion in autophagosome maturation, the biochemical details of which awaits to be further investigated.

## MEMBRANE-ASSOCIATED TECHNIQUES APPLIED IN AUTOPHAGY STUDIES

As autophagy is a biochemical process occurring on membrane or associated with membranes, techniques and assays developed to study membrane properties of organelles or membrane-associated proteins are required when studying the molecular mechanism of autophagy. Here, we summarized common techniques and assays to study membrane/organelle properties as showed in Table 3. In particular, we emphasized some successful applications in the study of autophagy.

**Table 3:** The summaries of biochemical technique/assays used to study properties of membrane in autophagy

Membrane Properties to study	Technique/assay to use		Principle and observable(s)	References
Lipid composition	Lipidomics of purified subcellular fraction by MS (Mass spectrometry)		MS of lipids from purified organelles	(Takamori et al., 2006; de la Ballina et al., 2020)
	IF (immunofluorescence) of specific PI probes		IF of specific PI targeting proteins	(Di Paolo and De Camilli, 2006)
Membrane tethering	DLS		Vesicle size change upon tethering or clustering	(Diao et al., 2015; Liu et al., 2016)
	EM		Morphology change of vesicles upon tethering or clustering.	(Diao et al., 2015)
	TIRF-based Single vesicle tethering assay		Overlap of fluorescence labeled vesicles	(Diao et al., 2015)
Lipid transfer	FRET based lipid transfer assay		FRET signal change upon lipid transfer	(Maeda et al., 2019)
Membrane fusion	Ensemble average assay (Bulk assay)	Lipid mixing assay: NBD/Rhod or DiI/DiD	FRET signal changes of FRET pairs upon lipid mixing	(Weber et al., 1998)
		Content mixing assay: SrB	Recover of self-quench high concentration fluorescence signal upon fusion	(Ma et al., 2013; Diao et al., 2015)
		Simultaneous lipid mixing and content mixing NBD/Marine blue and PhycoE and Cy5	FRET signal changes of two FRET pairs. One pair on the membrane, the other pair inside the vesicles.	(Zucchi and Zick, 2011; Liu et al., 2016; Liu et al., 2017)
	TIRF based Single vesicle fusion assay	Lipid mixing DiI/DiD	TIRF fluorescence signal changes	(Kyoung et al., 2011; Diao et al., 2012; Kyoung et al., 2013)
		Content mixing SrB or Cy5/Cy3		
Membrane scission	High resolution TEM		Visualize the closure under EM	(Zhen et al., 2019)
	Optogenetic closure assay Photo active release assay		Photo active to dissociation and association LOVTRAP tag reversibly to monitor the closure state of mitophagosome	(Zhen et al., 2019)
	HaloTag based IF assay		Utilizing membrane permeable and impermeable Halo tag fluorescent substrate, visualize closed autophagosome and unclosed phagophore	(Takahashi et al., 2018; Takahashi et al., 2019)
	Optical tweezer-based force test		Giant unilamellar vesicles pulled by optical tweezer and measure the force changing upon ATP release	(Schoneberg et al., 2018)
	Atomic Force Microscope based visualization of *in vitro* ESCRT-III contain membrane system		*In vitro* assemble ESCRT-III on lipid bilayers, and visualize it by Atomic Force Microscope	(Chiaruttini et al., 2015)

Lipid composition can affect membrane properties, such as curvature, surface charge, and binding affinity with certain proteins. There are many reviews discussing how lipid geometry and charge can affect membrane properties (de la Ballina et al., 2020). As it is important to determine lipid composition when studying the organelles, usually lipidomics by mass spectrometry is applied to purified subcellular fractions. This method has been applied successfully in many kinds of organelles that are easily purified, such as neuro vesicles from the brain tissue (Takamori et al., 2006). Lysosomes can be readily purified by protocols (Cudjoe Jr et al., 2017), but the other organelles relevant to autophagy are difficult to purify (de la Ballina et al., 2020). Recently, Martin Graef and his colleagues used anti-GFP (Green fluorescent protein)-magnetic beads to isolate the Atg8 vesicle membranes from 2GFP-Atg8 expressing yeast cells to study the lipidomics of autophagic membranes in detail, including the abundance of phospholipids with different head groups, chain length, and double bond numbers. They found that the Atg8-containing autophagic membrane contains a very high degree of desaturation lipids compared to other organelle membranes, which is conducive to highly dynamic phagophore properties. Besides, another way to verify the presence of particular phospholipids, such as different kinds of PI, is using their specific antibodies for immunofluorescence localization (Di Paolo and De Camilli, 2006). This might serve as a complementary approach when purifying certain organelles which are difficult to do, such as autolysosomes. Understanding the membrane composition of autophagic organelles more comprehensively can help us to understand the physiological functions of these organelles and to dissect their biochemical function during autophagy. Thus, improved techniques to purify autophagic organelles are required in the future.

The dynamic properties of membranes include lipid transfer, membrane fluidity, membrane tethering, membrane fusion, and membrane scission. Fluorescent-tagging and labeling are useful tools to be applied in these dynamic processes. For example, FRET (fluorescence resonance energy transfer) can occur between compatible fluorescent molecules and provide information about intermolecular distances. As previously discussed, lipid transfer is essential for membrane elongation. To investigate lipid transfer between two liposomes, a pair of FRET dyes can be engineered on one liposome and lipid transfer and efficiency can be calculated by measuring the fluorescence signal recovery rate of donor dye upon lipid transfer between the FRET-compatible liposomes and the blank liposomes. It’s worth mentioning that, the membrane fusion can also result in the change of FRET. The addition of dithionite after lipid transfer can be applied to differentiate these two cases. Dithionite oxidizes fluorescence dyes located on the outer leaflet of the lipid bilayers without affecting the dyes on the inner layer. So, the FRET donor signal will drop with the addition of dithionite during lipid transfer, but not for the case of vesicle fusion. Otomo et al. successfully applied this method to demonstrate the lipid transfer properties of ATG2A (Maeda et al., 2019). With FRET-based *in vivo* assays, the protein concentration needs to be of physiological relevance as high protein concentration might cause aggregation, liposome clustering, and delay of lipid transfer. A programmable DNA-origami platform can be applied to fix the distance between two liposomes and prevent the clustering of liposomes by proteins that tend to aggregate (Bian et al., 2019). As the direct visualization of *in vivo* lipid transfer has not yet been achieved, *in vitro* lipid transfer assays combined with the genetic studies became the primary method of assessing lipid transfer ability of the lipid transfer proteins. However, the reconstituted lipid transfer rate *in vitro* might be slow compared to the need of living cells. Studies to complete this lipid transfer pathway will emerge in large number. Once more ATG2-interaction proteins found, the *in vitro* lipid transfer assay will be applied to study how these proteins cooperate with each other to achieve the efficient lipid transfer in cells.

The membranes of autophagosomes and lysosomes are tethered before they fuse. How does the lysosome determine its tethering target in a membrane trafficking network? There are many unknown pieces in this puzzle, including Rab GTPases, GAPs (GTPase-activating protein), GEFs, and effector proteins (e.g., HOPS complex). How do these proteins cooperate to achieve the efficient and precise tethering between the autophagosome and lysosome? The membrane tethering assays no doubt can provide a good tool to study this regulation mechanism. First, tethering can be characterized by changes in vesicle size (Liu et al., 2016). Usually, the dramatic size changes detected with DLS (Dynamic light scattering) indicate vesicular tethering, but the possibility of vesicle fusion needs to be excluded. Usually, the resultant liposome size of two fused liposomes is smaller than the size of two tethered liposomes. Besides, proteolysis of tethered proteins can rescue the size change observed in DLS experiments, but not in the case of fusion. Second, the morphology observation by EM is a direct way to visualize the clustered vesicles. Third, TIRF (Total Internal Reflection Fluorescence) microscopy can distinguish the overlap of two vesicles with two different dyes. TIRF occurs within 100–200 nm of the cover glass, and TIRF observation can be achieved by tethering one liposome with biotin-PE to the cover glass through the Biotin-Streptavidin-Biotin interaction. With the combination of these three methods, Qing Zhong and his colleagues demonstrated that ATG14 can tether membranes and bridge autophagosomes and lysosomes to accelerate their fusion by SNAREs (Diao et al., 2015), and that this tethering ability is dependent on its oligomerization. It is worth emphasizing that single-vesicle experiments can reveal large heterogeneities at the single vesicle level, which are hidden in ensemble studies.

The fusion between autophagosomes and lysosomes makes the lysozymes and other enzymes accessible to substrates. This fusion represents a critical step in autophagy but is still a largely undefined process. Many proteins have been found to play a crucial role in this process via genetic analysis, however, how they coordinate with each other to achieve this fusion process remains elusive. To study membrane fusion, both bulk assays and TIRF-based single vesicle assays can be applied. The bulk assays are advantageous in that they do not require an expensive TIRF microscope, and can be set up to observe lipid mixing only, content mixing only, or simultaneous lipid and content mixing. It’s important to note that observations of lipid mixing signals does not necessarily represent a full fusion (Jun and Wickner, 2007). So, a content mixing assay in parallel is necessary when studying membrane fusion. By monitoring the FRET signal built up from a pair of FRET dyes separately encapsulated in previously distinct liposomes or by monitoring the fluorescence signal recovery from self-quenched dye encapsulated in one liposome, content mixing can be observed and quantified. Qing Zhong and his colleagues successfully applied the DiI/DiD-lipid mixing assay and self-quenched SrB (sulforhodamine B) content mixing assay to discover the tethering ability of ATG14 to promote STX17-SNAP29-VAMP8 driven fusion of autophagosomes and lysosomes (Diao et al., 2015). When using SrB as an indicator for content mixing in bulk assays, a careful control experiment needs to be set in parallel because SrB tends to leak from vesicles (Yu et al., 2013). Another content mixing assay first designed by Wickner to study yeast vacuole fusion can rule out the false-positive signals derived from leaking dyes (Zucchi and Zick, 2011). This assay has been applied successfully in the membrane fusion studies for neurotransmitter release, carried by the Rizo-Rey group (Liu et al., 2016; Liu et al., 2017; Sitarska et al., 2017; Xu et al., 2017). This presents an alternative method to be used for autophagic membrane fusion in the future. Besides, the TIRF-based single vesicle assays for membrane fusion with proper design can provide more information on individual vesicles compared to ensemble assays (Kyoung et al., 2011; Kyoung et al., 2013). The state of docking, hemifusion, and fusion can be distinguished from each other and the fusion rate of each docked vesicle pair can be monitored to get quantitative fusion rates of all the vesicles within the sample. This provides scientists a powerful tool to reveal the precise function of proteins and which step of membrane fusion they regulate. So far, the single-vesicle assays are more frequently used to study membrane fusion in neurology, however, one day it will likely be implemented into the autophagic membrane fusion field when more regulatory proteins are identified in autophagosome-lysosome fusion.

The antithesis of membrane fusion is membrane scission. Membrane scission plays an important role in many cellular activities, including cytokinetic abscission, plasma membrane vesicle budding, endosomal sorting, ER, Golgi compartment formation, autophagosome maturation, etc. Previously, *in vitro* membrane scission was successfully reconstituted by biochemists to study endosomal sorting. Using optical tweezers and an integrated confocal microscope, Johannes et al. measured the changing force and imaged the membrane nanotubes pulled by the ESCRT-III complex (Schoneberg et al., 2018), which biochemically proved that ESCRT-III-mediated membrane scission requires ATP and VPS4. Besides, by using atomic force microscopy and TEM (transmission electron microscopy), Nicolas et al. observed that ESCRT-III can polymerize into spirals springs at lipid bilayers and the relaxation of loaded ESCRT-III spiral springs can drive membrane deformation (Chiaruttini et al., 2015). Recently, membrane scission by ESCRT during autophagosome closure has been confirmed by imaging-based methods described in the previous paragraph. So far, autophagic membrane scission has not been reconstituted *in vitro*, however, as more autophagy-specific protein regulators in this scission reaction are identified, the application of this reconstitution assay will be required soon.

## DISCUSSION AND OUTSTANDING QUESTIONS

Autophagy is a conserved and essential degradation pathway in cells. It is not only a housekeeping mechanism to clean-up the aggregated proteins, damaged organelles, and pathogens, but also maintains cellular homeostasis by providing nutrition. Autophagy occurs on membranes and is driven by many membrane-associated proteins. With the discoveries and characterization of these proteinaceous regulators, we understand more about this important process. However, its molecular mechanism regarding membrane extension for phagophore elongation, membrane scission for autophagosome formation, and membrane tethering and fusion for autophagosome maturation are relatively unknown compared to vesicle-plasma membrane fusion for neurotransmitter release and vacuole fusion in yeast. Many questions remained to be answered. What are the autophagic-specific regulators of these membrane remodeling processes that ensure these reactions happen at the right time, at the proper membrane, and with the desired speed? What are the cell signals that trigger or impact these levels of regulation? How are these protein regulators transported? What is the biochemical mechanism behind these regulations? A combination of powerful screening assays like CRISPR-Cas9 or TAP-MS and genetic analysis could be a good start to find the answers.

Besides, the quantitative analysis in autophagy research is a trend that has grown in the past few decades. The research mainly focused on the qualitative analysis of autophagic regulators and which steps are rate-limiting steps in autophagy is still under debate. For autophagosome biogenesis, recruitment of autophagic machinery, synthesis, and transfer of phospholipids, phosphorylation of PIs, recognition, and binding of cargo substrates, shaping of membrane curvature, or phagophore membrane closure might be the rate-limiting steps. Whereas for autophagosome maturation, recruitment of tethering/fusion machinery, the activity of lysosomes, disassembly/priming of SNAREs, or substrates hydrolysis might be the rate-limiting steps. Furthermore, in different tissues or organisms, the rate-limiting steps in autophagy might be different. So, the development of innovative technology, quantitative analysis in autophagy is gradually emerging and badly required. Altogether, it will be critical to know how to measure autophagy and how it is precisely regulated, both for further study and better translational clinical application, like drug development, disease treatment, etc.

## References

[CR2] Abada A, Levin-Zaidman S, Porat Z, Dadosh T, Elazar Z (2017). SNARE priming is essential for maturation of autophagosomes but not for their formation. Proc Natl Acad Sci USA.

[CR3] Asao H, Sasaki Y, Arita T, Tanaka N, Endo K, Kasai H, Takeshita T, Endo Y, Fujita T, Sugamura K (1997). Hrs is associated with STAM, a signal-transducing adaptor molecule. Its suppressive effect on cytokine-induced cell growth. J Biol Chem.

[CR4] Axe EL, Walker SA, Manifava M, Chandra P, Roderick HL, Habermann A, Griffiths G, Ktistakis NT (2008). Autophagosome formation from membrane compartments enriched in phosphatidylinositol 3-phosphate and dynamically connected to the endoplasmic reticulum. J Cell Biol.

[CR5] Baba T, Toth DJ, Sengupta N, Kim YJ, Balla T (2019). Phosphatidylinositol 4,5-bisphosphate controls Rab7 and PLEKHM1 membrane cycling during autophagosome-lysosome fusion. EMBO J.

[CR6] Babst M, Katzmann DJ, Estepa-Sabal EJ, Meerloo T, Emr SD (2002). Escrt-III: an endosome-associated heterooligomeric protein complex required for mvb sorting. Dev Cell.

[CR7] Babst M, Katzmann DJ, Snyder WB, Wendland B, Emr SD (2002). Endosome-associated complex, ESCRT-II, recruits transport machinery for protein sorting at the multivesicular body. Dev Cell.

[CR8] Baskaran S, Ragusa MJ, Boura E, Hurley JH (2012). Two-site recognition of phosphatidylinositol 3-phosphate by PROPPINs in autophagy. Mol Cell.

[CR9] Bas L, Papinski D, Licheva M, Torggler R, Rohringer S, Schuschnig M, Kraft C (2018). Reconstitution reveals Ykt6 as the autophagosomal SNARE in autophagosome–vacuole fusion. J Cell Biol.

[CR10] Bean BD, Dziurdzik SK, Kolehmainen KL, Fowler CM, Kwong WK, Grad LI, Davey M, Schluter C, Conibear E (2018). Competitive organelle-specific adaptors recruit Vps13 to membrane contact sites. J Cell Biol.

[CR11] Behrends C, Sowa ME, Gygi SP, Harper JW (2010). Network organization of the human autophagy system. Nature.

[CR12] Besprozvannaya M, Dickson E, Li H, Ginburg KS, Bers DM, Auwerx J, Nunnari J (2018). GRAM domain proteins specialize functionally distinct ER-PM contact sites in human cells. Elife.

[CR13] Bian X, Zhang Z, Xiong Q, De Camilli P, Lin C (2019). A programmable DNA-origami platform for studying lipid transfer between bilayers. Nat Chem Biol.

[CR14] Bielli A, Haney CJ, Gabreski G, Watkins SC, Bannykh SI, Aridor M (2005). Regulation of Sar1 NH2 terminus by GTP binding and hydrolysis promotes membrane deformation to control COPII vesicle fission. The Journal of cell biology.

[CR15] Buchkovich NJ, Henne WM, Tang S, Emr SD (2013). Essential N-terminal insertion motif anchors the ESCRT-III filament during MVB vesicle formation. Dev Cell.

[CR17] Caillat C, Macheboeuf P, Wu Y, McCarthy AA, Boeri-Erba E, Effantin G, Gottlinger HG, Weissenhorn W, Renesto P (2015). Asymmetric ring structure of Vps4 required for ESCRT-III disassembly. Nat Commun.

[CR18] Carlsson SR, Simonsen A (2015). Membrane dynamics in autophagosome biogenesis. J Cell Sci.

[CR19] Carroll B, Mohd-Naim N, Maximiano F, Frasa MA, McCormack J, Finelli M, Thoresen SB, Perdios L, Daigaku R, Francis RE (2013). The TBC/RabGAP Armus coordinates Rac1 and Rab7 functions during autophagy. Dev Cell.

[CR20] Chang C, Young LN, Morris KL, von Bülow S, Schöneberg J, Yamamoto-Imoto H, Oe Y, Yamamoto K, Nakamura S, Stjepanovic G (2019). Bidirectional control of autophagy by BECN1 BARA domain dynamics. Mol Cell.

[CR21] Chan EY, Longatti A, McKnight NC, Tooze SA (2009). Kinase-inactivated ULK proteins inhibit autophagy via their conserved C-terminal domains using an Atg13-independent mechanism. Mol Cell Biol.

[CR22] Chen D, Fan W, Lu Y, Ding X, Chen S, Zhong Q (2012). A mammalian autophagosome maturation mechanism mediated by TECPR1 and the Atg12-Atg5 conjugate. Mol Cell.

[CR23] Chiaruttini N, Redondo-Morata L, Colom A, Humbert F, Lenz M, Scheuring S, Roux A (2015). Relaxation of loaded ESCRT-III spiral springs drives membrane deformation. Cell.

[CR24] Chowdhury S, Otomo C, Leitner A, Ohashi K, Aebersold R, Lander GC, Otomo T (2018). Insights into autophagosome biogenesis from structural and biochemical analyses of the ATG2A-WIPI4 complex. Proc Natl Acad Sci.

[CR25] Chung T (2019). How phosphoinositides shape autophagy in plant cells. Plant Sci.

[CR26] Cudjoe EK, Saleh T, Hawkridge AM, Gewirtz DA (2017). Proteomics insights into autophagy. Proteomics.

[CR27] Daum G, Vance JE (1997). Import of lipids into mitochondria. Prog Lipid Res.

[CR28] de la Ballina LR, Munson MJ, Simonsen A (2020). Lipids and lipid-binding proteins in selective autophagy. J Mol Biol.

[CR29] de Kroon AI, Dolis D, Mayer A, Lill R, de Kruijff B (1997). Phospholipid composition of highly purified mitochondrial outer membranes of rat liver and Neurospora crassa. Is cardiolipin present in the mitochondrial outer membrane?. Biochim Biophys Acta (BBA).

[CR30] Delorme-Axford E, Klionsky DJ (2018). Transcriptional and post-transcriptional regulation of autophagy in the yeast Saccharomyces cerevisiae. J Biol Chem.

[CR31] Diao J, Ishitsuka Y, Lee H, Joo C, Su Z, Syed S, Shin YK, Yoon TY, Ha T (2012). A single vesicle-vesicle fusion assay for in vitro studies of SNAREs and accessory proteins. Nat Protoc.

[CR32] Diao J, Liu R, Rong Y, Zhao M, Zhang J, Lai Y, Zhou Q, Wilz LM, Li J, Vivona S (2015). ATG14 promotes membrane tethering and fusion of autophagosomes to endolysosomes. Nature.

[CR33] Dikic I, Elazar Z (2018). Mechanism and medical implications of mammalian autophagy. Nat Rev Mol Cell Biol.

[CR34] Ding X, Jiang X, Tian R, Zhao P, Li L, Wang X, Chen S, Zhu Y, Mei M, Bao S (2019). RAB2 regulates the formation of autophagosome and autolysosome in mammalian cells. Autophagy.

[CR35] Di Paolo G, De Camilli P (2006). Phosphoinositides in cell regulation and membrane dynamics. Nature.

[CR36] Dove SK, Dong K, Kobayashi T, Williams FK, Michell RH (2009). Phosphatidylinositol 3,5-bisphosphate and Fab1p/PIKfyve underPPIn endo-lysosome function. Biochem J.

[CR37] Dudley LJ, Cabodevilla AG, Makar AN, Sztacho M, Michelberger T, Marsh JA, Houston DR, Martens S, Jiang X, Gammoh N (2019). Intrinsic lipid binding activity of ATG16L1 supports efficient membrane anchoring and autophagy. EMBO J.

[CR38] Ebner P, Poetsch I, Deszcz L, Hoffmann T, Zuber J, Ikeda F (2018). The IAP family member BRUCE regulates autophagosome–lysosome fusion. Nat Commun.

[CR39] Fan W, Nassiri A, Zhong Q (2011). Autophagosome targeting and membrane curvature sensing by Barkor/Atg14 (L). Proc Natl Acad Sci USA.

[CR40] Feng Q, Luo Y, Zhang XN, Yang XF, Hong XY, Sun DS, Li XC, Hu Y, Li XG, Zhang JF (2020). MAPT/Tau accumulation represses autophagy flux by disrupting IST1-regulated ESCRT-III complex formation: a vicious cycle in Alzheimer neurodegeneration. Autophagy.

[CR41] Fujioka Y, Noda NN, Nakatogawa H, Ohsumi Y, Inagaki F (2010). Dimeric coiled-coil structure of Saccharomyces cerevisiae Atg16 and its functional significance in autophagy. J Biol Chem.

[CR42] Fujioka Y, Alam JM, Noshiro D, Mouri K, Ando T, Okada Y, May AI, Knorr RL, Suzuki K, Ohsumi Y (2020). Phase separation organizes the site of autophagosome formation. Nature.

[CR43] Fujita N, Itoh T, Omori H, Fukuda M, Noda T, Yoshimori T (2008). The Atg16L complex specifies the site of LC3 lipidation for membrane biogenesis in autophagy. Mol Biol Cell.

[CR44] Gatica D, Lahiri V, Klionsky DJ (2018). Cargo recognition and degradation by selective autophagy. Nat Cell Biol.

[CR45] Gatta AT, Carlton JG (2019). The ESCRT-machinery: closing holes and expanding roles. Curr Opin Cell Biol.

[CR46] Ge L, Schekman R (2014). The ER-Golgi intermediate compartment feeds the phagophore membrane. Autophagy.

[CR47] Ge L, Melville D, Zhang M, Schekman R (2013). The ER–Golgi intermediate compartment is a key membrane source for the LC3 lipidation step of autophagosome biogenesis. Elife.

[CR48] Ge L, Zhang M, Schekman R (2014). Phosphatidylinositol 3-kinase and COPII generate LC3 lipidation vesicles from the ER-Golgi intermediate compartment. elife.

[CR49] Ge L, Zhang M, Kenny SJ, Liu D, Maeda M, Saito K, Mathur A, Xu K, Schekman R (2017). Remodeling of ER-exit sites initiates a membrane supply pathway for autophagosome biogenesis. EMBO Rep.

[CR50] Gómez-Sánchez R, Rose J, Guimarães R, Mari M, Papinski D, Rieter E, Geerts WJ, Hardenberg R, Kraft C, Ungermann C (2018). Atg9 establishes Atg2-dependent contact sites between the endoplasmic reticulum and phagophores. J Cell Biol.

[CR51] Graef M (2020) Recent advances in the understanding of autophagosome biogenesis. F1000Res 9. 10.12688/f1000research.22111.1 PMC7101016 32266060

[CR52] Graef M, Friedman JR, Graham C, Babu M, Nunnari J (2013). ER exit sites are physical and functional core autophagosome biogenesis components. Mol Biol Cell.

[CR53] Hailey DW, Rambold AS, Satpute-Krishnan P, Mitra K, Sougrat R, Kim PK, Lippincott-Schwartz J (2010). Mitochondria supply membranes for autophagosome biogenesis during starvation. Cell.

[CR54] Hanada T, Noda NN, Satomi Y, Ichimura Y, Fujioka Y, Takao T, Inagaki F, Ohsumi Y (2007). The Atg12-Atg5 conjugate has a novel E3-like activity for protein lipidation in autophagy. J Biol Chem.

[CR55] Hasegawa J, Iwamoto R, Otomo T, Nezu A, Hamasaki M, Yoshimori T (2016). Autophagosome-lysosome fusion in neurons requires INPP5E, a protein associated with Joubert syndrome. EMBO J.

[CR56] Hayashi-Nishino M, Fujita N, Noda T, Yamaguchi A, Yoshimori T, Yamamoto A (2009). A subdomain of the endoplasmic reticulum forms a cradle for autophagosome formation. Nat Cell Biol.

[CR57] He S, Ni D, Ma B, Lee J-H, Zhang T, Ghozalli I, Pirooz SD, Zhao Z, Bharatham N, Li B (2013). PtdIns (3) P-bound UVRAG coordinates Golgi–ER retrograde and Atg9 transport by differential interactions with the ER tether and the beclin 1 complex. Nat Cell Biol.

[CR58] Hollenstein DM, Kraft C (2020). Autophagosomes are formed at a distinct cellular structure. Curr Opin Cell Biol.

[CR59] Hosokawa N, Hara T, Kaizuka T, Kishi C, Takamura A, Miura Y, Iemura S, Natsume T, Takehana K, Yamada N (2009). Nutrient-dependent mTORC1 association with the ULK1-Atg13-FIP200 complex required for autophagy. Mol Biol Cell.

[CR60] Ho CY, Alghamdi TA, Botelho RJ (2012). Phosphatidylinositol-3,5-bisphosphate: no longer the poor PIP2. Traffic.

[CR61] Huang X, Sun S, Wang X, Fan F, Zhou Q, Lu S, Cao Y, Wang QW, Dong MQ, Yao J (2019). Mechanistic insights into the SNARE complex disassembly. Sci Adv.

[CR62] Hurley JH, Young LN (2017). Mechanisms of autophagy initiation. Annu Rev Biochem.

[CR63] Ichimura Y, Kirisako T, Takao T, Satomi Y, Shimonishi Y, Ishihara N, Mizushima N, Tanida I, Kominami E, Ohsumi M (2000). A ubiquitin-like system mediates protein lipidation. Nature.

[CR64] Ishihara N, Hamasaki M, Yokota S, Suzuki K, Kamada Y, Kihara A, Yoshimori T, Noda T, Ohsumi Y (2001). Autophagosome requires specific early Sec proteins for its formation and NSF/SNARE for vacuolar fusion. Mol Biol Cell.

[CR65] Itakura E, Kishi-Itakura C, Mizushima N (2012). The hairpin-type tail-anchored SNARE syntaxin 17 targets to autophagosomes for fusion with endosomes/lysosomes. Cell.

[CR66] Jang DJ, Lee JA (2016). The roles of phosphoinositides in mammalian autophagy. Arch Pharm Res.

[CR67] Jeynov B, Lay D, Schmidt F, Tahirovic S, Just WW (2006). Phosphoinositide synthesis and degradation in isolated rat liver peroxisomes. FEBS Lett.

[CR68] Jiang P, Nishimura T, Sakamaki Y, Itakura E, Hatta T, Natsume T, Mizushima N (2014). The HOPS complex mediates autophagosome-lysosome fusion through interaction with syntaxin 17. Mol Biol Cell.

[CR69] Johansen T, Lamark T (2020). Selective autophagy: ATG8 family proteins, LIR motifs and cargo receptors. J Mol Biol.

[CR70] Jung CH, Jun CB, Ro SH, Kim YM, Otto NM, Cao J, Kundu M, Kim DH (2009). ULK-Atg13-FIP200 complexes mediate mTOR signaling to the autophagy machinery. Mol Biol Cell.

[CR71] Jun Y, Wickner W (2007). Assays of vacuole fusion resolve the stages of docking, lipid mixing, and content mixing. Proc Natl Acad Sci.

[CR72] Kabeya Y, Mizushima N, Ueno T, Yamamoto A, Kirisako T, Noda T, Kominami E, Ohsumi Y, Yoshimori T (2000). LC3, a mammalian homologue of yeast Apg8p, is localized in autophagosome membranes after processing. EMBO J.

[CR73] Karanasios E, Stapleton E, Manifava M, Kaizuka T, Mizushima N, Walker SA, Ktistakis NT (2013). Dynamic association of the ULK1 complex with omegasomes during autophagy induction. J Cell Sci.

[CR74] Katzmann DJ, Babst M, Emr SD (2001). Ubiquitin-dependent sorting into the multivesicular body pathway requires the function of a conserved endosomal protein sorting complex, ESCRT-I. Cell.

[CR75] Kirisako T, Baba M, Ishihara N, Miyazawa K, Ohsumi M, Yoshimori T, Noda T, Ohsumi Y (1999). Formation process of autophagosome is traced with Apg8/Aut7p in yeast. J Cell Biol.

[CR76] Knaevelsrud H, Soreng K, Raiborg C, Haberg K, Rasmuson F, Brech A, Liestol K, Rusten TE, Stenmark H, Neufeld TP (2013). Membrane remodeling by the PX-BAR protein SNX18 promotes autophagosome formation. J Cell Biol.

[CR77] Komada M, Kitamura N (1995). Growth factor-induced tyrosine phosphorylation of Hrs, a novel 115-kilodalton protein with a structurally conserved putative zinc finger domain. Mol Cell Biol.

[CR78] Kostelansky MS, Schluter C, Tam YY, Lee S, Ghirlando R, Beach B, Conibear E, Hurley JH (2007). Molecular architecture and functional model of the complete yeast ESCRT-I heterotetramer. Cell.

[CR79] Kriegenburg F, Ungermann C, Reggiori F (2018). Coordination of autophagosome–lysosome fusion by ATG8 family members. Curr Biol.

[CR80] Kriegenburg F, Bas L, Gao J, Ungermann C, Kraft C (2019). The multi-functional SNARE protein Ykt6 in autophagosomal fusion processes. Cell Cycle.

[CR81] Ktistakis NT (2019). Who plays the ferryman: ATG2 channels lipids into the forming autophagosome. J Cell Biol.

[CR82] Kumar N, Leonzino M, Hancock-Cerutti W, Horenkamp FA, Li P, Lees JA, Wheeler H, Reinisch KM, De Camilli P (2018). VPS13A and VPS13C are lipid transport proteins differentially localized at ER contact sites. J Cell Biol.

[CR83] Kyoung M, Srivastava A, Zhang Y, Diao J, Vrljic M, Grob P, Nogales E, Chu S, Brunger AT (2011). In vitro system capable of differentiating fast Ca2+-triggered content mixing from lipid exchange for mechanistic studies of neurotransmitter release. Proc Natl Acad Sci.

[CR84] Kyoung M, Zhang Y, Diao J, Chu S, Brunger AT (2013). Studying calcium-triggered vesicle fusion in a single vesicle-vesicle content and lipid-mixing system. Nat Protoc.

[CR85] Lai LTF, Ye H, Zhang W, Jiang L, Lau WCY (2019). Structural biology and electron microscopy of the autophagy molecular machinery. Cells.

[CR86] Laraia L, Friese A, Corkery DP, Konstantinidis G, Erwin N, Hofer W, Karatas H, Klewer L, Brockmeyer A, Metz M (2019). The cholesterol transfer protein GRAMD1A regulates autophagosome biogenesis. Nat Chem Biol.

[CR87] Lemus L, Ribas JL, Sikorska N, Goder V (2016). An ER-localized SNARE protein is exported in specific COPII vesicles for autophagosome biogenesis. Cell Rep.

[CR88] Levine B, Kroemer G (2019). Biological functions of autophagy genes: a disease perspective. Cell.

[CR89] Liang C, Lee JS, Inn KS, Gack MU, Li Q, Roberts EA, Vergne I, Deretic V, Feng P, Akazawa C (2008). Beclin1-binding UVRAG targets the class C Vps complex to coordinate autophagosome maturation and endocytic trafficking. Nat Cell Biol.

[CR1] Liu X, Seven AB, Camacho M, Esser V, Xu J, Trimbuch T, Quade B, Su L, Ma C, Rosenmund C (2016). Functional synergy between the Munc13 C-terminal C1 and C2 domains. Elife.

[CR90] Liu X, Seven AB, Xu J, Esser V, Su L, Ma C, Rizo J (2017). Simultaneous lipid and content mixing assays for in vitro reconstitution studies of synaptic vesicle fusion. Nat Protoc.

[CR91] Li L, Zhong Q (2016). Autophagosome-lysosome fusion: PIs to the rescue. EMBO J.

[CR92] Lystad AH, Simonsen A (2016). Phosphoinositide-binding proteins in autophagy. FEBS Lett.

[CR93] Lystad AH, Carlsson SR, Laura R, Kauffman KJ, Nag S, Yoshimori T, Melia TJ, Simonsen A (2019). Distinct functions of ATG16L1 isoforms in membrane binding and LC3B lipidation in autophagy-related processes. Nat Cell Biol.

[CR94] Maeda S, Otomo C, Otomo T (2019). The autophagic membrane tether ATG2A transfers lipids between membranes. Elife.

[CR95] Maruyama T, Noda NN (2018). Autophagy-regulating protease Atg4: structure, function, regulation and inhibition. J Antibiot.

[CR96] Matsui T, Jiang P, Nakano S, Sakamaki Y, Yamamoto H, Mizushima N (2018). Autophagosomal YKT6 is required for fusion with lysosomes independently of syntaxin 17. J Cell Biol.

[CR97] Matsushita M, Suzuki NN, Obara K, Fujioka Y, Ohsumi Y, Inagaki F (2007). Structure of Atg5.Atg16, a complex essential for autophagy. J Biol Chem.

[CR98] Ma C, Su L, Seven AB, Xu Y, Rizo J (2013). Reconstitution of the vital functions of Munc18 and Munc13 in neurotransmitter release. Science.

[CR99] Ma M, Liu J-J, Li Y, Huang Y, Ta N, Chen Y, Fu H, Ye M-D, Ding Y, Huang W (2017). Cryo-EM structure and biochemical analysis reveal the basis of the functional difference between human PI3KC3-C1 and-C2. Cell Res.

[CR100] McEwan DG, Popovic D, Gubas A, Terawaki S, Suzuki H, Stadel D, Coxon FP, Miranda de Stegmann D, Bhogaraju S, Maddi K (2015). PLEKHM1 regulates autophagosome-lysosome fusion through HOPS complex and LC3/GABARAP proteins. Mol Cell.

[CR101] Melia TJ, Lystad AH, Simonsen A (2020). Autophagosome biogenesis: from membrane growth to closure. J Cell Biol.

[CR102] Mercer TJ, Gubas A, Tooze SA (2018). A molecular perspective of mammalian autophagosome biogenesis. J Biol Chem.

[CR103] Miao G, Zhang Y, Chen D, Zhang H (2020). The ER-localized transmembrane protein TMEM39A/SUSR2 regulates autophagy by controlling the trafficking of the PtdIns(4)P phosphatase SAC1. Mol Cell.

[CR104] Mizushima N (2007). Autophagy: process and function. Genes Dev.

[CR105] Mizushima N, Yoshimori T, Ohsumi Y (2011). The role of Atg proteins in autophagosome formation. Annu Rev Cell Dev Biol.

[CR106] Moreau K, Ravikumar B, Renna M, Puri C, Rubinsztein DC (2011). Autophagosome precursor maturation requires homotypic fusion. Cell.

[CR107] Muñoz-Braceras S, Calvo R, Escalante R (2015). TipC and the chorea-acanthocytosis protein VPS13A regulate autophagy in Dictyostelium and human HeLa cells. Autophagy.

[CR108] Nair U, Jotwani A, Geng J, Gammoh N, Richerson D, Yen W-L, Griffith J, Nag S, Wang K, Moss T (2011). SNARE proteins are required for macroautophagy. Cell.

[CR109] Naito T, Ercan B, Krshnan L, Triebl A, Koh DHZ, Wei F-Y, Tomizawa K, Torta FT, Wenk MR, Saheki Y (2019). Movement of accessible plasma membrane cholesterol by the GRAMD1 lipid transfer protein complex. eLife.

[CR110] Nakamura S, Yoshimori T (2017). New insights into autophagosome–lysosome fusion. J Cell Sci.

[CR111] Nakatogawa H (2020) Mechanisms governing autophagosome biogenesis. Nat Rev Mol Cell Biol 10.1038/s41580-020-0241-0 32372019

[CR112] Nakatogawa H, Ichimura Y, Ohsumi Y (2007). Atg8, a ubiquitin-like protein required for autophagosome formation, mediates membrane tethering and hemifusion. Cell.

[CR113] Nascimbeni AC, Codogno P, Morel E (2017). Phosphatidylinositol-3-phosphate in the regulation of autophagy membrane dynamics. FEBS J.

[CR114] Nath S, Dancourt J, Shteyn V, Puente G, Fong WM, Nag S, Bewersdorf J, Yamamoto A, Antonny B, Melia TJ (2014). Lipidation of the LC3/GABARAP family of autophagy proteins relies on a membrane-curvature-sensing domain in Atg3. Nat Cell Biol.

[CR115] Nishimura T, Tooze SA (2020). Emerging roles of ATG proteins and membrane lipids in autophagosome formation. Cell Discov.

[CR116] Odorizzi G, Babst M, Emr SD (1998). Fab1p PtdIns(3)P 5-kinase function essential for protein sorting in the multivesicular body. Cell.

[CR117] Ogawa M, Yoshikawa Y, Kobayashi T, Mimuro H, Fukumatsu M, Kiga K, Piao Z, Ashida H, Yoshida M, Kakuta S (2011). A Tecpr1-dependent selective autophagy pathway targets bacterial pathogens. Cell Host Microbe.

[CR118] Omari S, Makareeva E, Roberts-Pilgrim A, Mirigian L, Jarnik M, Ott C, Lippincott-Schwartz J, Leikin S (2018). Noncanonical autophagy at ER exit sites regulates procollagen turnover. Proc Natl Acad Sci.

[CR119] Osawa T, Noda NN (2019). Atg2: A novel phospholipid transfer protein that mediates de novo autophagosome biogenesis. Protein Sci.

[CR120] Osawa T, Alam JM, Noda NN (2019). Membrane-binding domains in autophagy. Chem Phys Lipids.

[CR121] Osawa T, Ishii Y, Noda NN (2019). Human ATG2B possesses a lipid transfer activity which is accelerated by negatively charged lipids and WIPI4. Genes Cells.

[CR122] Osawa T, Kotani T, Kawaoka T, Hirata E, Suzuki K, Nakatogawa H, Ohsumi Y, Noda NN (2019). Atg2 mediates direct lipid transfer between membranes for autophagosome formation. Nat Struct Mol Biol.

[CR123] Otomo T, Maeda S (2019). ATG2A transfers lipids between membranes in vitro. Autophagy.

[CR124] Otomo T, Chowdhury S, Lander GC (2018). The rod-shaped ATG2A-WIPI4 complex tethers membranes in vitro. Contact.

[CR125] Palamiuc L, Ravi A, Emerling BM (2020). Phosphoinositides in autophagy: current roles and future insights. FEBS J.

[CR126] Pankiv S, Clausen TH, Lamark T, Brech A, Bruun JA, Outzen H, Overvatn A, Bjorkoy G, Johansen T (2007). p62/SQSTM1 binds directly to Atg8/LC3 to facilitate degradation of ubiquitinated protein aggregates by autophagy. J Biol Chem.

[CR127] Petiot A, Ogier-Denis E, Blommaart EF, Meijer AJ, Codogno P (2000). Distinct classes of phosphatidylinositol 3′-kinases are involved in signaling pathways that control macroautophagy in HT-29 cells. J Biol Chem.

[CR128] Polson HE, de Lartigue J, Rigden DJ, Reedijk M, Urbé S, Clague MJ, Tooze SA (2010). Mammalian Atg18 (WIPI2) localizes to omegasome-anchored phagophores and positively regulates LC3 lipidation. Autophagy.

[CR129] Preiss R (2017) Autophagy gene overexpression in *Saccharomyces cerevisiae* for accelerated sparkling wine production 10.1007/s00253-018-9304-y 30120525

[CR130] Puri C, Renna M, Bento CF, Moreau K, Rubinsztein DC (2013). Diverse autophagosome membrane sources coalesce in recycling endosomes. Cell.

[CR131] Ragusa MJ, Stanley RE, Hurley JH (2012). Architecture of the Atg17 complex as a scaffold for autophagosome biogenesis. Cell.

[CR132] Ravikumar B, Moreau K, Jahreiss L, Puri C, Rubinsztein DC (2010). Plasma membrane contributes to the formation of pre-autophagosomal structures. Nat Cell Biol.

[CR133] Raymond CK, Howald-Stevenson I, Vater CA, Stevens TH (1992). Morphological classification of the yeast vacuolar protein sorting mutants: evidence for a prevacuolar compartment in class E vps mutants. Mol Biol Cell.

[CR134] Reggiori F, Ungermann C (2017). Autophagosome maturation and fusion. J Mol Biol.

[CR135] Reggiori F, Shintani T, Chong H, Nair U, Klionsky DJ (2005). Atg9 cycles between mitochondria and the pre-autophagosomal structure in yeasts. Autophagy.

[CR136] Romanov J, Walczak M, Ibiricu I, Schuchner S, Ogris E, Kraft C, Martens S (2012). Mechanism and functions of membrane binding by the Atg5-Atg12/Atg16 complex during autophagosome formation. EMBO J.

[CR137] Rong Y, Liu M, Ma L, Du W, Zhang H, Tian Y, Cao Z, Li Y, Ren H, Zhang C (2012). Clathrin and phosphatidylinositol-4,5-bisphosphate regulate autophagic lysosome reformation. Nat Cell Biol.

[CR138] Russell RC, Tian Y, Yuan H, Park HW, Chang YY, Kim J, Kim H, Neufeld TP, Dillin A, Guan KL (2013). ULK1 induces autophagy by phosphorylating Beclin-1 and activating VPS34 lipid kinase. Nat Cell Biol.

[CR139] Rusten TE, Stenmark H (2009). How do ESCRT proteins control autophagy?. J Cell Sci.

[CR140] Schoneberg J, Lee IH, Iwasa JH, Hurley JH (2017). Reverse-topology membrane scission by the ESCRT proteins. Nat Rev Mol Cell Biol.

[CR141] Schoneberg J, Pavlin MR, Yan S, Righini M, Lee IH, Carlson LA, Bahrami AH, Goldman DH, Ren X, Hummer G (2018). ATP-dependent force generation and membrane scission by ESCRT-III and Vps4. Science.

[CR142] Schütter M, Giavalisco P, Brodesser S, Graef M (2020). Local fatty acid channeling into phospholipid synthesis drives phagophore expansion during autophagy. Cell.

[CR143] Schu PV, Takegawa K, Fry MJ, Stack JH, Waterfield MD, Emr SD (1993). Phosphatidylinositol 3-kinase encoded by yeast VPS34 gene essential for protein sorting. Science.

[CR144] Shatz O, Holland P, Elazar Z, Simonsen A (2016). Complex relations between phospholipids, autophagy, and neutral lipids. Trends Biochem Sci.

[CR145] Shibutani ST, Yoshimori T (2014). A current perspective of autophagosome biogenesis. Cell Res.

[CR146] Shima T, Kirisako H, Nakatogawa H (2019). COPII vesicles contribute to autophagosomal membranes. J Cell Biol.

[CR147] Shintani T, Suzuki K, Kamada Y, Noda T, Ohsumi Y (2001). Apg2p functions in autophagosome formation on the perivacuolar structure. J Biol Chem.

[CR148] Sitarska E, Xu J, Park S, Liu X, Quade B, Stepien K, Sugita K, Brautigam CA, Sugita S, Rizo J (2017). Autoinhibition of Munc18-1 modulates synaptobrevin binding and helps to enable Munc13-dependent regulation of membrane fusion. Elife.

[CR149] Slessareva JE, Routt SM, Temple B, Bankaitis VA, Dohlman HG (2006). Activation of the phosphatidylinositol 3-kinase Vps34 by a G protein α subunit at the endosome. Cell.

[CR150] Soreng K, Munson MJ, Lamb CA, Bjorndal GT, Pankiv S, Carlsson SR, Tooze SA, Simonsen A (2018). SNX18 regulates ATG9A trafficking from recycling endosomes by recruiting Dynamin-2. EMBO Rep.

[CR151] Stadel D, Millarte V, Tillmann KD, Huber J, Tamin-Yecheskel BC, Akutsu M, Demishtein A, Ben-Zeev B, Anikster Y, Perez F (2015). TECPR2 cooperates with LC3C to regulate COPII-dependent ER export. Mol cell.

[CR152] Stroupe C, Collins KM, Fratti RA, Wickner W (2006). Purification of active HOPS complex reveals its affinities for phosphoinositides and the SNARE Vam7p. EMBO J.

[CR153] Sun Q, Fan W, Chen K, Ding X, Chen S, Zhong Q (2008). Identification of Barkor as a mammalian autophagy-specific factor for Beclin 1 and class III phosphatidylinositol 3-kinase. Proc Natl Acad Sci.

[CR154] Sun Q, Zhang J, Fan W, Wong KN, Ding X, Chen S, Zhong Q (2011). The RUN domain of rubicon is important for hVps34 binding, lipid kinase inhibition, and autophagy suppression. J Biol Chem.

[CR155] Suzuki H, Osawa T, Fujioka Y, Noda NN (2017). Structural biology of the core autophagy machinery. Curr Opin Struct Biol.

[CR156] Takahashi Y, Coppola D, Matsushita N, Cualing HD, Sun M, Sato Y, Liang C, Jung JU, Cheng JQ, Mul JJ (2007). Bif-1 interacts with Beclin 1 through UVRAG and regulates autophagy and tumorigenesis. Nat Cell Biol.

[CR157] Takahashi Y, Meyerkord CL, Hori T, Runkle K, Fox TE, Kester M, Loughran TP, Wang HG (2011). Bif-1 regulates Atg9 trafficking by mediating the fission of Golgi membranes during autophagy. Autophagy.

[CR158] Takahashi Y, He H, Tang Z, Hattori T, Liu Y, Young MM, Serfass JM, Chen L, Gebru M, Chen C (2018). An autophagy assay reveals the ESCRT-III component CHMP2A as a regulator of phagophore closure. Nat Commun.

[CR159] Takahashi Y, Liang X, Hattori T, Tang Z, He H, Chen H, Liu X, Abraham T, Imamura-Kawasawa Y, Buchkovich NJ (2019). VPS37A directs ESCRT recruitment for phagophore closure. J Cell Biol.

[CR160] Takamori S, Holt M, Stenius K, Lemke EA, Gronborg M, Riedel D, Urlaub H, Schenck S, Brugger B, Ringler P (2006). Molecular anatomy of a trafficking organelle. Cell.

[CR161] Tang Z, Takahashi Y, He H, Hattori T, Chen C, Liang X, Chen H, Young MM, Wang HG (2019). TOM40 targets Atg2 to mitochondria-associated ER membranes for phagophore expansion. Cell Rep.

[CR162] Thorburn A (2018). Autophagy and disease. J Biol Chem.

[CR163] Tong J, Manik MK, Im YJ (2018). Structural basis of sterol recognition and nonvesicular transport by lipid transfer proteins anchored at membrane contact sites. Proc Natl Acad Sci.

[CR164] Tsuboyama K, Koyama-Honda I, Sakamaki Y, Koike M, Morishita H, Mizushima N (2016). The ATG conjugation systems are important for degradation of the inner autophagosomal membrane. Science.

[CR165] Tsukada M, Ohsumi Y (1993). Isolation and characterization of autophagy-defective mutants of Saccharomyces cerevisiae. FEBS Lett.

[CR166] Valverde DP, Yu S, Boggavarapu V, Kumar N, Lees JA, Walz T, Reinisch KM, Melia TJ (2019). ATG2 transports lipids to promote autophagosome biogenesis. J Cell Biol.

[CR167] Velikkakath AK, Nishimura T, Oita E, Ishihara N, Mizushima N (2012). Mammalian Atg2 proteins are essential for autophagosome formation and important for regulation of size and distribution of lipid droplets. Mol Biol Cell.

[CR168] Wang K, Yang Z, Liu X, Mao K, Nair U, Klionsky DJ (2012). Phosphatidylinositol 4-kinases are required for autophagic membrane trafficking. J Biol Chem.

[CR169] Wang Z, Miao G, Xue X, Guo X, Yuan C, Wang Z, Zhang G, Chen Y, Feng D, Hu J (2016). The Vici syndrome protein EPG5 is a Rab7 effector that determines the fusion specificity of autophagosomes with late endosomes/lysosomes. Mol Cell.

[CR170] Watanabe Y, Kobayashi T, Yamamoto H, Hoshida H, Akada R, Inagaki F, Ohsumi Y, Noda NN (2012). Structure-based analyses reveal distinct binding sites for Atg2 and phosphoinositides in Atg18. J Biol Chem.

[CR171] Weber T, Zemelman BV, McNew JA, Westermann B, Gmachl M, Parlati F, Söllner TH, Rothman JE (1998). SNAREpins: minimal machinery for membrane fusion. Cell.

[CR172] Wetzel L, Blanchard S, Rama S, Beier V, Kaufmann A, Wollert T (2020). TECPR1 promotes aggrephagy by direct recruitment of LC3C autophagosomes to lysosomes. Nat Commun.

[CR173] Wherrett JR, Huterer S (1972). Enrichment of bis-(monoacylglyceryl) phosphate in lysosomes from rat liver. J Biol Chem.

[CR174] White KI, Zhao M, Choi UB, Pfuetzner RA, Brunger AT (2018). Structural principles of SNARE complex recognition by the AAA+ protein NSF. Elife.

[CR175] Wickner W, Rizo J (2017). A cascade of multiple proteins and lipids catalyzes membrane fusion. Mol Biol Cell.

[CR176] Wollert T, Hurley JH (2010). Molecular mechanism of multivesicular body biogenesis by ESCRT complexes. Nature.

[CR177] Xie Z, Nair U, Klionsky DJ (2008). Atg8 controls phagophore expansion during autophagosome formation. Mol Biol Cell.

[CR178] Xu Z, Yang L, Xu S, Zhang Z, Cao Y (2015). The receptor proteins: pivotal roles in selective autophagy. Acta Biochim Biophys Sin.

[CR179] Xu J, Camacho M, Xu Y, Esser V, Liu X, Trimbuch T, Pan YZ, Ma C, Tomchick DR, Rosenmund C (2017). Mechanistic insights into neurotransmitter release and presynaptic plasticity from the crystal structure of Munc13-1 C1C2BMUN. Elife.

[CR180] Yamamoto H, Kakuta S, Watanabe TM, Kitamura A, Sekito T, Kondo-Kakuta C, Ichikawa R, Kinjo M, Ohsumi Y (2012). Atg9 vesicles are an important membrane source during early steps of autophagosome formation. J Cell Biol.

[CR181] Ylä-Anttila P, Vihinen H, Jokitalo E, Eskelinen E-L (2009). 3D tomography reveals connections between the phagophore and endoplasmic reticulum. Autophagy.

[CR182] Yorikawa C, Shibata H, Waguri S, Hatta K, Horii M, Katoh K, Kobayashi T, Uchiyama Y, Maki M (2005). Human CHMP6, a myristoylated ESCRT-III protein, interacts directly with an ESCRT-II component EAP20 and regulates endosomal cargo sorting. Biochem J.

[CR183] Yu Z-Q, Ni T, Hong B, Wang H-Y, Jiang F-J, Zou S, Chen Y, Zheng X-L, Klionsky DJ, Liang Y (2012). Dual roles of Atg8− PE deconjugation by Atg4 in autophagy. Autophagy.

[CR184] Yu H, Rathore SS, Lopez JA, Davis EM, James DE, Martin JL, Shen J (2013). Comparative studies of Munc18c and Munc18-1 reveal conserved and divergent mechanisms of Sec1/Munc18 proteins. Proc Natl Acad Sci USA.

[CR185] Yu L, Chen Y, Tooze SA (2018). Autophagy pathway: cellular and molecular mechanisms. Autophagy.

[CR186] Zambrano F, Fleischer S, Fleischer B (1975). Lipid composition of the Golgi apparatus of rat kidney and liver in comparison with other subcellular organelles. Biochim Biophys Acta (BBA).

[CR187] Zhang X, Wang L, Ireland SC, Ahat E, Li J, Bekier ME, Zhang Z, Wang Y (2019). GORASP2/GRASP55 collaborates with the PtdIns3K UVRAG complex to facilitate autophagosome-lysosome fusion. Autophagy.

[CR188] Zhang A, Meng Y, Li Q, Liang Y (2020) The ESCRT complex negatively regulates Erg6 degradation under specific glucose restriction conditions. Traffic 10.1111/tra.12732 32378292

[CR189] Zhao YG, Zhang H (2019). Autophagosome maturation: an epic journey from the ER to lysosomes. J Cell Biol.

[CR190] Zhao M, Wu S, Zhou Q, Vivona S, Cipriano DJ, Cheng Y, Brunger AT (2015). Mechanistic insights into the recycling machine of the SNARE complex. Nature.

[CR191] Zhen Y, Spangenberg H, Munson MJ, Brech A, Schink KO, Tan KW, Sorensen V, Wenzel EM, Radulovic M, Engedal N (2019). ESCRT-mediated phagophore sealing during mitophagy. Autophagy.

[CR192] Zhou F, Wu Z, Zhao M, Murtazina R, Cai J, Zhang A, Li R, Sun D, Li W, Zhao L (2019). Rab5-dependent autophagosome closure by ESCRT. J Cell Biol.

[CR193] Zinser E, Daum G (1995). Isolation and biochemical characterization of organelles from the yeast, Saccharomyces cerevisiae. Yeast.

[CR194] Zucchi PC, Zick M (2011). Membrane fusion catalyzed by a Rab, SNAREs, and SNARE chaperones is accompanied by enhanced permeability to small molecules and by lysis. Mol Biol Cell.

